# Cortical Dynamics in Presence of Assemblies of Densely Connected Weight-Hub Neurons

**DOI:** 10.3389/fncom.2017.00052

**Published:** 2017-06-22

**Authors:** Hesam Setareh, Moritz Deger, Carl C. H. Petersen, Wulfram Gerstner

**Affiliations:** ^1^Laboratory of Computational Neuroscience, School of Computer and Communication Sciences and Brain Mind Institute, School of Life Sciences, École Polytechnique Fédérale de LausanneLausanne, Switzerland; ^2^Faculty of Mathematics and Natural Sciences, Institute for Zoology, University of CologneCologne, Germany; ^3^Laboratory of Sensory Processing, Brain Mind Institute, School of Life Sciences, École Polytechnique Fédérale de LausanneLausanne, Switzerland

**Keywords:** connectivity, hub neuron, neural assembly, spike frequency adaptation, up-state/down-state oscillation

## Abstract

Experimental measurements of pairwise connection probability of pyramidal neurons together with the distribution of synaptic weights have been used to construct randomly connected model networks. However, several experimental studies suggest that both wiring and synaptic weight structure between neurons show statistics that differ from random networks. Here we study a network containing a subset of neurons which we call weight-hub neurons, that are characterized by strong inward synapses. We propose a connectivity structure for excitatory neurons that contain assemblies of densely connected weight-hub neurons, while the pairwise connection probability and synaptic weight distribution remain consistent with experimental data. Simulations of such a network with generalized integrate-and-fire neurons display regular and irregular slow oscillations akin to experimentally observed up/down state transitions in the activity of cortical neurons with a broad distribution of pairwise spike correlations. Moreover, stimulation of a model network in the presence or absence of assembly structure exhibits responses similar to light-evoked responses of cortical layers in optogenetically modified animals. We conclude that a high connection probability into and within assemblies of excitatory weight-hub neurons, as it likely is present in some but not all cortical layers, changes the dynamics of a layer of cortical microcircuitry significantly.

## Introduction

Is it possible to uniquely constrain a model network of point neurons with experimental data? First, suppose that we have access to experimental measurements of electrophysiological properties of single neurons. Indeed, a wealth of single-neuron data exists (Markram et al., 2004, 2015) and methods have been developed that enable a rapid and reliable extraction of parameters of generalized integrate-and-fire neuron models from such experimental data (Jolivet et al., 2006; Pillow et al., 2008; Mensi et al., 2012; Pozzorini et al., 2013, 2015). Thus, parameters of neuron models, including spread of parameters caused by heterogeneity, can be completely constrained by experiments. Second, suppose that we have access to experimental measurements of the distribution of synaptic weights. Indeed, experimental data suggests a unimodal, possibly log-normal, distribution of EPSP amplitudes (Feldmeyer et al., 1999, 2002, 2006; Song et al., 2005; Frick et al., 2008; Lefort et al., 2009). Thus, we can constrain the distribution of synaptic weights in a network model with the data collected over many pairs of neurons. Third, suppose that we know the probability that two neurons (say, of types A and B located in layers n and m of the same cortical column) make a short-range connection from A to B. Again, such data exists (Lefort et al., 2009; Avermann et al., 2012) and should be used to constrain a network model. But is data collected on single neurons and pairs of neurons sufficient to constrain the parameters of a network model?

The answer is negative. There are at least two reasons: (i) The distribution of synaptic weights does not indicate whether a single neuron is driven by a random combination of strong and weak synapses, or whether one neuron receives all the strong input synapses and another one all the weak ones. Similarly, (ii) an average connection probability of say, 20 percent, is consistent with a network of a 1000 neurons where each neuron receives exactly 200 connections, but also equally consistent with a network where half the neurons receive 100 inputs and the other half 300. In this paper, we systematically explore network variants that implement the variations indicated under (i) and (ii) while keeping all single-neuron parameters, number of neurons, as well as pairwise connection probabilities and synaptic weight distributions fixed. To keep the arguments as transparent, consistent, and precise as possible, we focus on a single cortical layer of mouse barrel cortex and use data from a single lab (Lefort et al., 2009; Avermann et al., 2012).

The hypothetical variations (i) and (ii) make our networks different from a classical Erdős–Rényi random network. Indeed, experimental data from various labs indicate non-random features in network connectivity (Song et al., 2005; Yoshimura et al., 2005; Kampa et al., 2006; Perin et al., 2011). The influence of some of these features on activity patterns in neuronal networks has already been studied in a set of modeling papers (Koulakov et al., 2009; Roxin, 2011; Litwin-Kumar and Doiron, 2012; Hu et al., 2013, 2014; Pernice et al., 2013; Vasquez et al., 2013; Jahnke et al., 2014; Luccioli et al., 2014; McDonnell and Ward, 2014; Mazzucato et al., 2015, 2016). We introduce two features of network connectivity which we call degree-hub and weight-hub. These concern two types of neurons and are made based on the hypothetical variations (i) and (ii), respectively. In particular, if a few neurons receive more synaptic input connections than others (Roxin, 2011; Pernice et al., 2013; Tomm et al., 2014), we will refer to these neurons as degree-hubs. On the other hand, even in a network where there are no degree-hubs, there can still be a few neurons (which we will call weight-hubs) that receive all the strong connections while others receive all the weak connections (Koulakov et al., 2009; Tomm et al., 2014), but chosen such that the total distribution of synaptic weights across the network remains consistent with experimental data. More generally, such non-random features can be described as “correlations” in the connectivity matrix or synaptic weight distribution. For example, the input connectivity in a network with weight-hubs is correlated: It is more likely to find a second strong input connection in a neuron in which you have already found a strong synapse than in a neuron for which you have found a weak synapse.

Several experiments studied the existence of both degree-hub and weight-hub neurons in different regions of the brain. In the hippocampus GABAergic neurons that receive more synapses than average were detected (Bonifazi et al., 2009). Excitatory neurons which receive many synapses from inhibitory neurons were found in mouse frontal cortex (Fino and Yuste, 2011). Recently, Okun et al. (2015) found that neurons that are strongly correlated to the population-averaged firing rate receive larger numbers of synapses from their neighbors. Neurons receiving stronger connections from several other neurons were observed in experiments (Song et al., 2005; Perin et al., 2011). Yassin et al. (2010) and Cossart et al. (2003) investigated neocortical excitatory neurons that systematically fire more than other neurons. Such a high firing rate can be due to different intrinsic neuronal properties or more frequent or stronger excitatory synapses onto the neurons. In the latter case, the receiving neurons can be considered as candidates of degree- or weight-hubs. Even though experimental evidence for the existence of weight-hubs within pyramidal neurons is not yet convincing, we explore here signatures of hypothetical weight-hub neurons in neuronal activity.

Another experiment unravels a related but different phenomenon in the cortex. Yoshimura et al. (2005) suggest that excitatory neurons in the cortex can form assemblies and that neurons inside each assembly share common synaptic input. Here we explore a hypothetical network where connectivity between weight-hub neurons is higher than average. We show that such an elevated connection probability *between* weight-hub neurons significantly changes the dynamics of the network. Note that a subnetwork of densely connected weight-hub neurons can be interpreted as a neuronal assembly (Hebb, 1949).

We build a neuronal network which models layer 5A (L5) of a mouse barrel cortex. Neuron numbers, pairwise connection probabilities, and distribution of synaptic weights are matched to experimental data (Lefort et al., 2009; Avermann et al., 2012). Parameters of the neuron model such as membrane time constants, firing threshold and adaptation have been extracted from experimental data (Mensi et al., 2012). Our model is able to generate up-state/down-state oscillations (Steriade et al., 1993; Cowan and Wilson, 1994; Lampl et al., 1999). We show that in our network of adaptive integrate-and-fire neurons, the existence of weight-hub neurons is not sufficient for producing metastable up- and down-states. For oscillations to appear, weight-hub neurons need to form assemblies with dense internal connectivity. Another phenomenon that we address here is the different light-evoked responses of cortical supra-granular and infra-granular layers. Experiments (Beltramo et al., 2013) show that optogenetic stimulation in L5 leads to a large depolarization and a notable number of emitted spikes in non-stimulated neurons. In contrast after stimulation of a group of neurons in layer 2/3 (L2/3), non-stimulated neurons do not show significant responses. We show that such a difference can be explained by the presence or absence of an assembly of hub neurons. We hypothesize that in L5 weight-hub neurons are connected together densely and form assemblies while in L2/3 their connections are sparse. This may explain experimental observations (Sakata and Harris, 2009; Chauvette et al., 2010; Beltramo et al., 2013) which indicate that up-states are initiated in L5, and that L2/3 largely follows the oscillation passively.

## Materials and methods

### Neuron model and population parameters

In each simulation we model one cortical layer, either layer 5A (L5) or layer 2/3 (L2/3) from mouse somatosensory cortex. Based on experimental data (Lefort et al., 2009), our model of a L5 barrel column contains 454 excitatory and 90 inhibitory neurons while L2/3 contains 1691 excitatory and 230 inhibitory neurons. The two layers are studied separately and are not connected to each other.

As a neuron model, we use a current-based Generalized Integrate-and-Fire (GIF) model (Mensi et al., 2012) that features both an adaptation current and a dynamic threshold for spike-frequency adaptation. The GIF model parameters that we use in our simulations have been previously extracted from experimental data (Mensi et al., 2012). Importantly, the GIF model has been shown to capture with high accuracy both the subthreshold dynamics of the membrane potential and the spiking activity recorded from neurons in mouse barrel cortex slices during current injection (Mensi et al., 2012; Pozzorini et al., 2013). In this model, the subthreshold membrane potential *V*(*t*) is described by the differential equation:

(1) $$\begin{array}{r} C\frac{dV\left(t\right)}{dt}=-g_{L}\left(V\left(t\right)-E_{L}\right)-\underset{{\hat{t}}_{j}<t}{\sum }\eta \left(t-{\hat{t}}_{j}\right)+I\left(t\right) \end{array}$$

where the parameters *C*, *g*_*L*_ and *E*_*L*_ define the passive properties of the neuron (for parameter values see Table 1), *I*(*t*) is the input current and $\left\{\;{\hat{t}}_{j}\right\}$ are the spike times. Each time a spike is emitted, an intrinsic current with stereotypical shape η(*t*) is triggered (see Table 2). Currents triggered by different spikes accumulate and produce spike-frequency adaptation. Immediately after firing, the membrane potential is reset to *V*_reset_, integration of Equation (1) restarts and the neuron goes through an absolute refractory period of duration *t*_ref_.

**Table 1:** The mean of GIF neuron model parameters extracted from Mensi et al. (2012).

**Parameter**	**Excitatory**	**Inhibitory**
*C*(pF)	83.1	46.1
*g*_L_(nS)	3.7	6.6
*E*_L_(*mV*)	−67.0	−71.2
τ_ref_(ms)	4.0	4.0
*V*_reset_(*mV*)	−36.7	−48.4
η(*t*)	η_1_(*t*)+η_2_(*t*)	η_1_(*t*)+η_2_(*t*)
η_1_(*t*) (pA)	56.7*e*^−*t*/57.8ms^	31.8*e*^−*t*/11.5ms^
η_2_(*t*) (pA)	−6.9*e*^−*t*/218.2ms^	1.6*e*^−*t*/500.1ms^
γ(*t*)	γ_1_(*t*)+γ_2_(*t*)	γ_1_(*t*)+γ_2_(*t*)
γ_1_(*t*) (*mV*)	11.7*e*^−*t*/53.8ms^	5.6*e*^−*t*/11.5ms^
γ_2_(*t*) (*mV*)	1.8*e*^−*t*/640.0ms^	0.6*e*^−*t*/473.7ms^
λ_0_ (kHz)	10	10
△*V* (*mV*)	1.4	0.6
V_*__T_(*mV*)	−39.6	−41.2

*In all simulation except Figure 3A we use these parameter values with variations of ±15%, which are independently and uniformly distributed. In Figure 3A there is no variation of parameters*.

**Table 2:** Network parameters as extracted from mouse barrel cortex (Lefort et al., 2009; Avermann et al., 2012).

	**Connection Probability**	**τ_syn_(ms)**	**Synaptic weight**			
			**PSP amplitude (mV)**		***w_ij_* (pA)**	
			**Mean**	**Std**	**Mean**	**Std**
exc 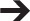 exc	19%	16.3	0.66	0.76	7.9	9.1
exc 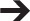 inh	37%	6.9	0.55	0.51	9.9	9.2
inh 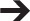 exc	50%	1.3	0.48	0.44	36.5	33.5
inh 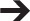 inh	35%	6.9	0.48	0.49	8.7	8.9

Spikes are produced stochastically according to a point process with the firing intensity

(2) $$\begin{array}{r} \lambda \left(t\right)=\lambda_{0}\text{exp}\left(\frac{V\left(t\right)-V_{T}\left(t\right)}{△V}\right) \end{array}$$

where λ_0_ is the stochastic intensity at the firing threshold *V*_*T*_, △*V* is a constant which defines the level of stochasticity and *V*_*T*_ is a time-dependent firing threshold:

(3) $$\begin{array}{r} V_{T}\left(t\right)=V_{T}^{*}+\underset{{\hat{t}}_{j}<t}{\sum }\gamma \left(t-{\hat{t}}_{j}\right) \end{array}$$

where $V_{T}^{*}$ is a constant and γ(*t*) describes the stereotypical time course of the firing threshold after the emission of an action potential (see Table 1).

For all neuronal parameters, we use the values given in Table 1 with ±15% uniformly distributed variations in all simulations, except for **Figure 3A**. For comparison in **Figure 3A** all neuron parameters are as in Table 1 without any variation. The values of Table 1 are extracted from experimental data from mouse barrel cortex (Mensi et al., 2012) and no parameter tuning of neuronal parameters was done for the network simulations reported here.

In the network, the input current *I*_*i*_(*t*) in Equation (1) is generated by synaptic current pulses into a specific neuron *i*

(4) $$\begin{array}{r} I_{i}\left(t\right)=\underset{j}{\sum }w_{ij}\underset{f}{\sum }\alpha \left(t-{\hat{t}}_{f}\right)=\underset{j}{\sum }w_{ij}\int_{0}^{\infty }\alpha \left(t\right)S_{j}\left(t-s\right)ds \end{array}$$

where $t_{j}^{f}$ is the *f*th spike of a presynaptic neuron *j* and $S_{j}=\sum_{f}\delta (t-{\hat{t}}_{f})$ is the spike train of neuron *j* where δ denotes the Dirac δ-function. We choose an exponential shape for post-synaptic currents (PSC) α with a time constant τ_syn_: $\alpha \left(t\right)=e^{-\left(t-△\right)/\tau_{\text{syn}}}$ for *t* ≥ Δ. The transmission delay (Δ) of synaptic connections in all our simulations is 1 ms. The symbol *w*_*ij*_ denotes the synaptic weight from neuron *j* to neuron *i*. The term synaptic weight is commonly used for either of two different quantities, either the amplitude of the PSC or the amplitude of the post-synaptic potential (PSP). In this study we take the first definition, i.e., *w*_*ij*_ denotes the PSC amplitude; see Equation 4. However, the experimental datasets we used report the synaptic weight based on the second definition (PSP amplitude). Given the neuronal parameters, one can easily relate the two quantities. We report the synaptic weight we used in our simulations according to both of the definitions in Table 2.

All network parameters (Table 2), e.g., connection probabilities, the distribution of synaptic weights and number of neurons are chosen based on previously published data extracted from mouse barrel cortex (Lefort et al., 2009; Avermann et al., 2012). At present there is no comparable dataset for L5 inhibitory neurons. Therefore, for inhibitory neurons, we use neuronal and network parameters similar to those of L2/3 inhibitory neurons (Avermann et al., 2012). Since, these neurons do not play a crucial role in initiating the up/down oscillations and stimulus-evoked responses, the exact choice of their parameters does not strongly affect the results. Moreover, the study of Pfeffer et al. (2013) explored some aspects of connectivity pattern between different subsets of inhibitory neurons and highlighted more similarities than differences between L2/3 and L5 inhibitory network. In the model, all neurons receive external Poisson noise whose properties are described in Table 3.

**Table 3:** Parameters of the external Poisson noise.

	**γ_Poisson_(Hz)**	**τ_syn_(ms)**	**Synaptic weight**
			***w_ij_* (pA)**	
Poisson 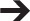 assembly1	100	16.3	30
Poisson 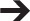 assembly2	100	16.3	30
Poisson 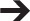 assembly3	100	16.3	30
Poisson 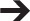 non-hubs	100	16.3	10
Poisson 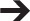 inhibitory	100	6.9	80

*Each neuron receives input from an independent Poisson process with a rate of r_Poisson_ and a synaptic weight w_ij_*.

In order to reproduce the light-evoked stimulation (**Figure 4**), we randomly select 15% of the neurons and inject a step current with amplitude 100 pA for 300 ms. For simulating the light-evoked response in L2/3, the connection probability and the mean synaptic weights inside excitatory population are 16.8% and 0.37 mV, respectively (Avermann et al., 2012). The other network parameters are the same as L5. For simulating the active cortical state (**Figure 6**), during the active period each neuron receives synaptic input from 70 Poisson process neurons firing with a rate of 5 Hz. The synaptic weights of synapses from Poisson neurons to assemblies, non-hubs and inhibitory neurons are 25, 5, and -25 pA, respectively.

All simulations were run using the Brian simulator (Goodman and Brette, 2008).

### Partitioning the excitatory population into weight-hubs and non-hubs subpopulations

**Figure 1:**
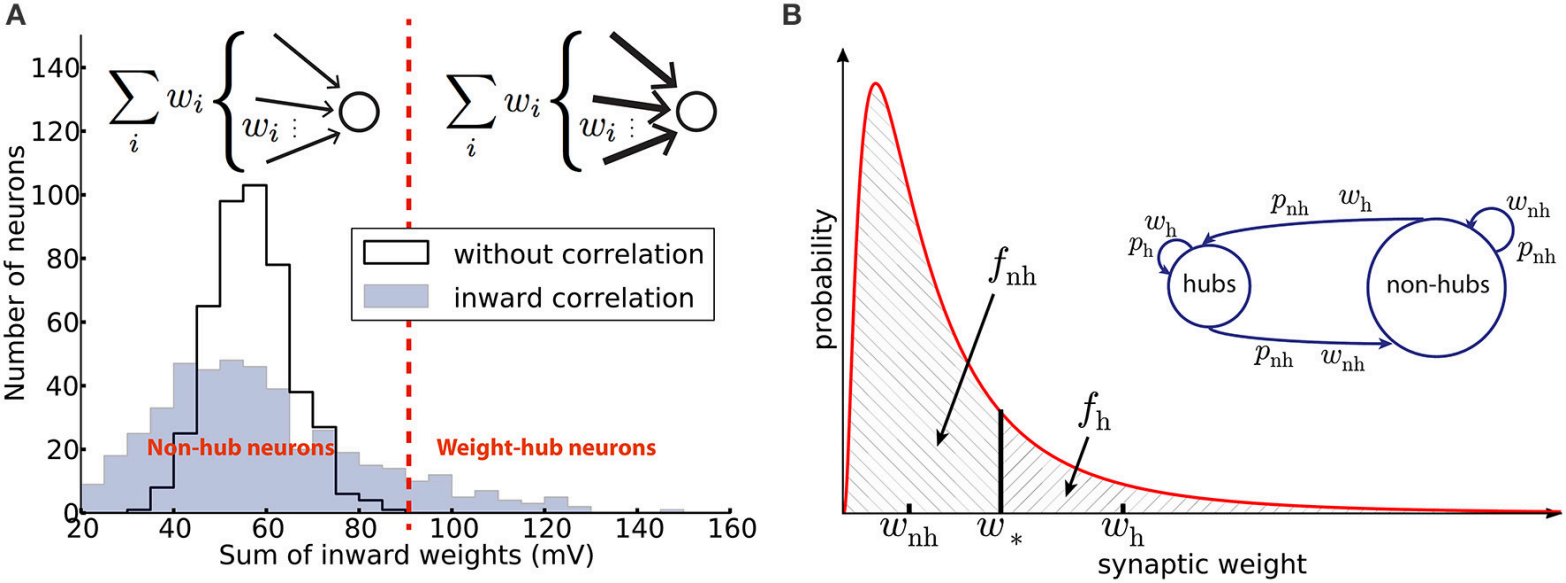
Networks with weight-hub neurons. **(A)** Histogram of the sum of inward weights for a random (solid line, network without weight-hubs) and inward correlated (filled, network with weight-hubs) network topology. While the random topology (without weight-hubs) shows an approximately normal distribution, the inward-correlated topology has a broader, lognormal-like distribution. Weight-hub neurons form the tail of this distribution. Both networks, without and with weight-hubs, have the same lognormal distribution of individual weights shown in **(B)** (red line). Inset: In a heterogeneous network with inward correlations, most neurons receive many weak (thin arrows) connections (top) whereas weight-hub neurons (bottom) receive many strong connections. **(B)** Fitting the experimental distribution (red line) of synaptic weights (EPSP amplitudes) by a two-element “homogeneous” distribution (dashed areas). The lognormal distribution (solid line) was fitted to experimental data (Lefort et al., 2009) and is used to find the values of weak and strong weights, *w*_nh_ and *w*_h_, respectively. Inset: Splitting the excitatory population into two subpopulations. Weight-hub neurons receive strong synaptic weights (*w*_h_) and non-hub neurons receive weak synaptic weights (wnh). All connection probabilities are low (*p*_nh_; nh: non-hub) except for the hub-to-hub connections (*p*_h_).

In order to distinguish between weight-hub and other neurons (“non-hubs”), and to capture the properties of weight-hub neuron subpopulations (assemblies), we use two methods explained in the following. The first method (heterogeneity approach) maintains the heterogeneity of synaptic weights in the population. The experimentally obtained probability density function of synaptic weights *p*(*w*) is well-approximated by a lognormal distribution (Lefort et al., 2009):

(5) $$\begin{array}{r} p\left(w\right)=\frac{1}{w\sigma \sqrt{2p}}e^{-{\left(\text{ln}\frac{w}{w_{\text{m}}}-\mu \right)}^{2}/2\sigma^{2}} \end{array}$$

where μ and *s* are the two parameters of the distribution and *w*_m_ is the median of synaptic weights (Figure 1).

Following Tomm et al. (2014), we first generate a synaptic weight matrix with local inward weight correlations, as described in the following. We start by generating an initial random connectivity matrix (connection probability *p* = 19%, see Table 2) with weights drawn from Equation (5). Let Ŵ = [_ŵ_*ij*_]*N*×*N*_ be the initial weight matrix, where *N* is the total number of excitatory neurons. We continue by generating a vector *A* = [_*a*_*i*_]*N*×1_ using another lognormal distribution. Now, we define a new weight matrix *W* by

(6) $$\begin{array}{r} w_{ij}={ŵ}_{ij}a_{i} \end{array}$$

A high value of *a*_*i*_ increases the weight of all synapses received by the *i*-th neuron. Therefore, a high value of *a*_*i*_ tends to convert the neuron to a weight-hub neuron, because it will have many large inward weights. Moreover, since multiplication of two lognormal variables yields a lognormal variable, we can be sure that new weights *w*_*ij*_ are drawn from a lognormal distribution. We choose the parameters of the two lognormal distributions (μ = 0.141, *s* = 0.924 and *w*_m_ = 0.372 mV for the distribution of ŵ_*ij*_, and μ = 1.4·10^−4^, *s* = 0.15 and *a*_m_ = 1 (median) for the distribution of *a*_*i*_) to set the mean and variance of the final weights equal to the values found in the experimental data (Lefort et al., 2009) (μ = 0.005, *s* = 0.936 and *w*_m_ = 0.419 mV). The mean weight is then 0.66 mV and the standard deviation is 0.76 mV with the above choice of parameters. *N*_h_ = 95 neurons out of *N*_e_ = 454 excitatory neurons (20.9%) were labeled as “weight-hubs,” by choosing those with the highest sum of inward weights (Figure 1A). Note that other values may have been used for the distribution parameters of ŵ_*ij*_ and *a*_*i*_, as long as the distribution of *w*_*ij*_ matches the experimental data (Lefort et al., 2009). We chose the mentioned parameters to achieve biologically plausible network dynamics (skewed firing rate distribution and low correlations between neurons). Note that if we only consider weight matrix Ŵ (without multiplying by *a*_*i*_), then our network does not contain weight-hub neurons. We used this approach in **Figure 4C**.

In a second step we rewire the network to increase the number of connections between weight-hub neurons in the assembly (such that connection probability between weight-hub neurons increases from $\bar{p}=19\%$ to *p*_h_ = 50%), while keeping the total number of connections fixed. To do so, we randomly select two unconnected weight-hub neurons and add a connection between them. The weight of this new synapse is again drawn from the lognormal distribution described earlier. Then we randomly select a connected neuron pair which contains at least one non-hub neuron and remove the synapse connecting them. This procedure is repeated until we reach the desired connection probability between weight-hubs. An issue here is that, since we remove a weak weight and add a strong one, the overall average weight increases slightly. However, the number of replaced synapses is very small compared to the overall number of synapses: The number of synapses between weight-hub neurons before the rewiring is $S_{\text{h}}^{\text{initial}}=N_{\text{h}}^{2}\bar{p}={\left(20.9\%\;\cdot \;N_{\text{e}}\right)}^{2}\bar{p}$ and after that it should be $S_{\text{h}}^{\text{final}}=N_{\text{h}}^{2}p_{\text{h}}={\left(20.9\%\;\cdot \;N_{\text{e}}\right)}^{2}p_{\text{h}}$. Therefore, the fraction of replaced synapses equals:

(7) $$\begin{array}{r} \frac{S_{\text{h}}^{\text{final}}-S_{\text{h}}^{\text{initial}}}{S_{\text{e}}}={\left(20.9\text{\%}\right)}^{2}\left(\frac{p_{\text{h}}}{\bar{p}}-1\right)=0.071 \end{array}$$

where $S_{\text{e}}=N_{\text{e}}^{2}\bar{p}$ is the total number of synapses between excitatory neurons. That means the rewiring concerns only 7.1% of all excitatory synapses, and therefore causes only a small increase of the average weight (average exc. weight is 0.659 mV before and is 0.666 mV after rewiring). Note that choosing a higher fraction of assembly neurons in the network increases the fraction of replaced synapses. For example, if assembly neurons form 35% of all excitatory neurons, 20% of all excitatory synapses will be affected. Clearly, it leads to a significant change in the average of excitatory weighs (0.659 mV before and 0.701 mV after rewiring).

The rewiring procedure can be modified in order to make several connected weight-hub assemblies instead of just one. To this end, we randomly assign weight-hub neurons into several groups. Then new synapses are added inside the groups and the same number of existing synapses between the groups or between a pair of non-hub neurons are removed. Using this procedure, each group becomes an assembly of densely connected weight-hub neurons.

As we observed in the previous method, we could choose different levels of heterogeneity in synaptic weight structure by choosing different values for the distribution parameters of ŵ_*ij*_ and *a*_*i*_. However, this heterogeneity does not affect the main outcome of the model, which is oscillation. We support this idea by showing that a model with homogenous weight structure is able to generate oscillations. Here we explain the second method for building weight-hub neurons in the excitatory population. This method produces homogenous synaptic weights within each subpopulation. Hence, we call it homogenous approach. We use it for the analytical results and for **Figure 3A**. The method splits the excitatory population into two subpopulations. The first one (the assembly of densely connected weight-hubs) contains *N*_h_ weight-hub neurons, the second one contains *N*_nh_ non-hub neurons. Weight-hubs are those neurons that receive strong synapses from other weight-hubs and from other excitatory neurons; all input weights onto weight-hubs have the same value *w*_h_. Non-hubs receive weak synapses, all with identical input weights *w*_nh_. Let us assume that all connection probabilities between and inside subpopulations are the same (*p*_nh_) except for the weight-hub to weight-hub connection probability (*p*_h_) which is larger. Figure 1A summarizes the parameters of this network structure.

The experimental data (Lefort et al., 2009) do not distinguish between weight-hubs and non-hubs but reports an overall synaptic weight distribution (*p*(*w*)) and an average connection probability ($\bar{p}$). We adjust the network parameters of the two homogeneous subpopulations using these data. The average connection probability ($\bar{p}$) should be maintained, despite the split of the population into weight-hubs and non-hubs. Computing the average connection probability in this model yields the equation:

(8) $$\begin{array}{r} \bar{p}=\frac{N_{\text{h}}^{2}p_{\text{h}}+N_{\text{nh}}^{2}p_{\text{nh}}+2N_{\text{h}}N_{\text{nh}}p_{\text{nh}}}{{\left(N_{\text{h}}+N_{\text{nh}}\right)}^{2}} \end{array}$$

We approximate the synaptic weight distribution *p*(*w*) obtained by experiments (Lefort et al., 2009) with a two-element distribution formed by *w*_h_ and *w*_nh_ (Figure 1A). The strategy is simple: a classification boundary (w_*_) divides the synaptic weights into two disjoint sets, i.e., synaptic weights lower than w_*_ and synaptic weights higher than w_*_. All weights lower than w_*_ are set to *w*_nh_ and the others to *w*_h_.

In order to obtain the value of w_*_, we introduce the fraction of weak connections *f*_nh_ as a parameter:

(9) $$\begin{array}{r} f_{\text{nh}}=\frac{N_{\text{nh}}^{2}p_{\text{nh}}+N_{\text{h}}N_{\text{nh}}p_{\text{nh}}}{{\left(N_{\text{h}}+N_{\text{nh}}\right)}^{2}\bar{p}} \end{array}$$

The classification boundary w_*_ follows from the condition that the probability mass of weak connections must account for the fraction of weak connections:

(10) $$\begin{array}{r} \int_{0}^{w_{*}}p\left(w\right)dw=f_{\text{nh}} \end{array}$$

Because *p*(*w*) is positive there is a unique solution for w_*_ which we determine numerically. Once the boundary w_*_ is fixed, averaging *w* over the respective support yields the synaptic weights of weight-hubs *w*_h_ and non-hubs *w*_nh_:

(11) $$\begin{array}{r} w_{\text{nh}}=\frac{1}{f_{\text{nh}}}\int_{0}^{w_{*}}p\left(w\right)dw \end{array}$$

(12) $$\begin{array}{r} w_{\text{h}}=\frac{1}{f_{\text{h}}}\int_{w_{*}}^{\infty }p\left(w\right)dw \end{array}$$

where *f*_h_ = 1−*f*_nh_. A similar procedure may be applied in the case of several connected weight-hub neurons subpopulations. Choosing *p*_h_ = 50% and *N*_h_ = 95, we obtain the remaining parameter values: *w*_h_ = 1.42 mV, *w*_nh_ = 0.34 mV for PSP amplitudes, and *p*_nh_ = 18%. The PSC amplitudes can be calculated using the PSP amplitudes: *w*_h_ = 16.9 pA and *w*_nh_ = 4.0 pA.

### Rate-current relations

Consider a population or a subpopulation of neurons. We can obtain a relation between the average firing rate of all neurons and the average synaptic input current using two different approaches. The first approach employs the neurons' gain function, a generalization of the frequency-current (*f*−*I*) curve (the terms firing frequency and firing rate are used interchangeably here). Injecting a weakly fluctuating current *I*_syn_ into a neuron causes an average firing rate of

(13) $$\begin{array}{r} r=g\left(\langle I_{\text{syn}}\rangle ,\sigma_{\text{I}}\right) \end{array}$$

where *g* is the gain function and 〈*I*_syn_〉 and *s*_I_ are the average and standard deviation of synaptic current over time, respectively. Although there are ways to compute the firing rate of adaptive integrate-and-fire neuron models in closed-form (Fourcaud-Trocmé et al., 2003; Hertäg et al., 2014) or by using a self-consistent numerical approach (La Camera et al., 2004; Richardson, 2007, 2009), we obtain it here by numerical simulations, using a certain amount of fluctuations in the input:

(14) $$\begin{array}{r} I\left(t\right)=\langle I_{\text{syn}}\rangle +\frac{\sigma_{\text{I}}}{\sqrt{q_{2}}}\int_{0}^{\infty }\alpha^{2}\left(\sigma \right)\xi \left(t-\sigma \right)ds \end{array}$$

where α(*t*) is the shape of an elementary postsynaptic current (PSC) defined in Equation (4), ξ(*t*) is white noise of unit standard deviation and $q_{2}=\int_{0}^{\infty }\alpha^{2}\left(t\right)dt$. If the current is injected for short episodes of 10 ms or less, we can estimate the firing rate in the non-adapted state by averaging over several trials. If it is injected for a longer time, we can divide the time into intervals of 10 ms and extract the frequency-current relation in the different, progressively more adapted states.

The second relation between the average firing rate and the average synaptic current follows from the network activity; see Amit and Brunel (1997) and Gerstner et al. (2014). Each neuron *i* receives the synaptic current produced by the input spike train:

(15) $$\begin{array}{r} I_{i,syn}\left(t\right)=\underset{j}{\sum }w_{ij}\left(\int_{0}^{\infty }\alpha \left(s\right)S_{j}\left(t-s\right)ds\right) \end{array}$$

where *S*_*j*_(*t*) is the spike train of *j*-th neuron, and *w*_*ij*_ is the synaptic weight of this input onto the receiving neuron. By averaging both sides over time and input neurons we obtain the average input current (also known as the mean field) $\langle I_{\text{syn}}\rangle =Npq\bar{w}r$, where *N* and *p* are the number of neurons in the population and the connection probability between them, respectively. Here $q=\int_{0}^{\infty }\alpha \left(t\right)dt$ is the total charge of one PSC pulse, $\bar{w}$ is the average synaptic weight and *r* is the average firing rate of neurons in this population. Rearranging this equation yields the network feedback relation:

(16) $$\begin{array}{r} r=\frac{\langle I_{\text{syn}}\rangle }{Npq\bar{w}} \end{array}$$

which is a linear relation of 〈*I*_syn_〉 and *r* with slope $1/Npq\bar{w}$. We refer to the denominator as the network feedback (*C*_fb_) of the population:

(17) $$\begin{array}{r} C_{\text{fb}}=Npq\bar{w} \end{array}$$

Equations (13, 17) will be used in the “Results” Section to get insight into the network dynamics.

### K-means clustering method

K-means is a machine learning method for assigning data samples of a dataset to K clusters. In this method, each data sample is represented by a vector of numbers.

The algorithm works as follows. It initializes the center vectors of the K clusters randomly. Then, K clusters are created by assigning each data sample to the nearest center vector (using Euclidean distance). Afterward, the new center of each cluster is calculated by averaging over all data samples to the cluster. The algorithm repeats the assignment and averaging steps until it converges (i.e., until no change happens to the clusters by repeating these steps).

An important issue is how to determine the number of clusters (K) to begin with. This has generally to be done with the task in mind. Here we use the algorithm for two tasks. The first one is distinguishing weight-hubs from non-hubs. Clearly, in this task K = 2, because we are looking for two different classes of neurons. The second task is assigning weight-hubs to different clusters. Here, we use the so-called elbow method for choosing the value of K: We run the algorithm for different values of K (2, 3, …). Generally, the error of clustering (sum of squared distances between each data sample and the center of its cluster) decreases with increasing K. However, we choose the K at which the error decreases abruptly and a greater K does not decrease the error that much. This method leads us to K = 3 for the second task.

## Results

### Layer 5-model network produces irregular oscillations

Both during anesthesia and slow-wave sleep cortical neurons show slow oscillations (~1 Hz) between two states (Steriade et al., 1993; Cowan and Wilson, 1994; Lampl et al., 1999; Sanchez-Vives and McCormick, 2000; Sanchez-Vives et al., 2000; Petersen et al., 2003), the active up-state and the quiescent down-state. The underlying mechanism of this phenomenon is not fully understood, but several neuronal network models have been suggested, mostly based on short-term plasticity (Holcman and Tsodyks, 2006; Melamed et al., 2008; Ghorbani et al., 2012).

Here we model cortical L5 with neuron model parameters and network parameters extracted from experimental data of a single column of somatosensory cortex in mice (see Section Materials and Methods and Tables 1, 2). An important feature of our model is that its excitatory population (which consists of 454 neurons) contains three assemblies of densely (*p* = 50%) connected weight-hubs which consist of 45, 30, and 20 weight-hub neurons, respectively. A weight-hub neuron, or simply weight-hub, is defined here as a neuron receiving many strong synapses so that the sum of incoming synaptic weights across all connections from other neurons in the network is large compared to that of other neurons (Figure 1A).

**Figure 2:**
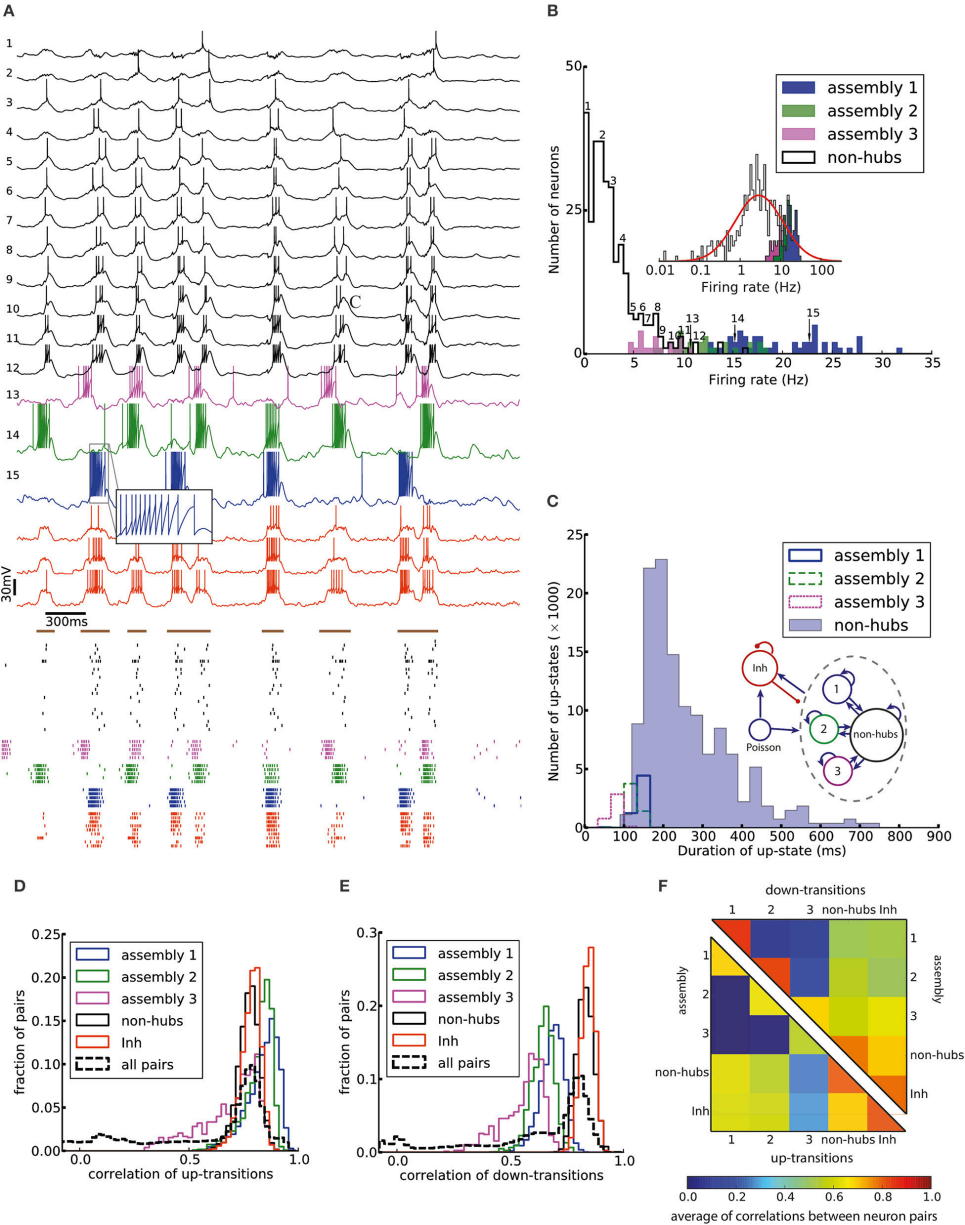
Irregular up- and down-state transitions in a network with three assemblies of densely connected weight-hubs. **(A-Top)** Membrane potential of sample non-hub (black, labeled 1–12) and weight-hub (labeled 13–15) and inhibitory neurons (red, without label). Inside each group neurons have been sorted by their firing rate. Brown bars indicate time intervals which are considered as up-states for bottommost inhibitory neuron. **(A-Bottom)** Raster plot of several neurons of each population (same color). **(B)** Rate distribution of excitatory neurons. Numbered labels indicate the firing rate of neurons whose membrane potential traces are shown in **(A)**. Inset: The distribution of firing rates is close to a normal distribution (red curve) on a (semi-) logarithmic scale. **(C)** Histograms of the up-state duration for each group of excitatory neurons. The coefficients of variation for the assemblies are 0.06, 0.10, and 0.16, which signifies regular durations. The non-hub neurons (filled histogram) exhibit a broad distribution of up-state durations with coefficient of variation of 0.42. Inset: The Excitatory population contains three assemblies of weight-hubs and a large population of non-hubs. **(D,E)** Distribution of pairwise Pearson correlation coefficients of transition times from down- to up-state **(D)** and from up- to down-state **(E)** inside each subpopulation (solid lines) and over all 145,530 pairs of neurons (dashed lines). Transitions of two neurons are counted as coincident if they happen in the same time bin of 20 ms. **(F)** Averaged Pearson correlation coefficients of transitions from down- to up-state (upper triangle) and up- to down-state (lower triangle).

Figure 2A shows the membrane potential and spike raster of several sample neurons. Simulations show that the model exhibits irregular up/down state transitions reminiscent of irregular slow oscillations in anesthetized cortex (Stern et al., 1997; Lampl et al., 1999). In order to compare the up-state durations in the model with experimental data (Stern et al., 1997; Cossart et al., 2003), we quantify the variability of the duration T of the up-state by the coefficient of variation, defined as std(T)/mean(T), where the up-state duration T is measured as the duration for which the membrane potential of a neuron stays at least 10 mV above the resting potential (*E*_L_). To this end, the membrane potential is smoothed by filtering with a Gaussian filter kernel (of width 20 ms) in order to remove rapid fluctuations. The coefficient of variation of this presumed up-state duration, averaged over all neurons that have not been classified as weight-hub, is 0.42, which shows that their up-state duration is rather irregular (Figure 2C). Similarly, if we repeatedly select 10 excitatory neurons (choosing randomly from both weight-hubs and other neurons) and measure the average coefficient of variation of the up-state duration, we find a coefficient of variation of 0.40 ± 0.06.

The L5-network model produces a skewed and long tailed distribution of firing rates in the whole population (Figure 2B) that is approximately lognormal (Figure 2B, inset). Weight-hub neurons have a high firing rate and form the tail of the distribution, whereas non-hubs have a low firing rate. The overall shape of the firing rate distribution is consistent with observations in *in-vivo* experiments (Hromádka et al., 2008; Vijayan et al., 2010).

We investigated the correlations of transition times from down- to up-state and vice versa (Figures 2D–F). Transitions inside subpopulations are highly correlated. The mean correlation coefficient for transition from down- to up-state is 0.84, 0.82, 0.69 for neurons within assemblies 1, 2, and 3, respectively, 0.77 for non-hub neurons and 0.78 for inhibitory neurons (Student's *t*-test for difference in mean: all *p*-values are smaller than 10^−10^). The mean cross-correlation for all pairs of neurons is 0.58, indicating a high correlation between randomly chosen pairs of neurons. The corresponding values for transition from up- to down-state are 0.68, 0.64, 0.56 for assemblies 1, 2, and 3, respectively, 0.83 for non-hub neurons, 0.84 for inhibitory neurons and 0.60 for all pairs of neurons (Student's *t*-test for difference in mean: all *p*-values are smaller than 10^−10^). These results indicate that an overall synchrony between neurons in the up-/down state oscillations is maintained, consistent with recordings from multiple extracellular electrodes (Petersen et al., 2003; Fucke et al., 2011). Note that assemblies oscillate out of phase, but not in anti-phase, because they do not strongly compete with each other, as opposed to a network in a winner-takes-all mode. Therefore, occasionally more than one assembly is active at a time (see raster plot in Figure 2A). Competitive neurons with anti-phase oscillations would instead lead to reduced correlation of up/down state transitions, averaged across all neuron pairs in the network. High correlations inside each subpopulation also increase the overall correlation. The peak of the overall correlation distribution mostly belongs to correlations of pairs inside subpopulations (Figure 2B). Another noteworthy point is that since the size of assembly 3 is small (20 neurons), its properties differ from other assemblies. In particular, the firing rate of assembly 3 neurons is less than that of the two other assemblies, and even less than several non-hub neurons (Figure 2B). Also, the correlation of up/down transitions is less than other assemblies and non-hub neurons.

**Figure 3:**
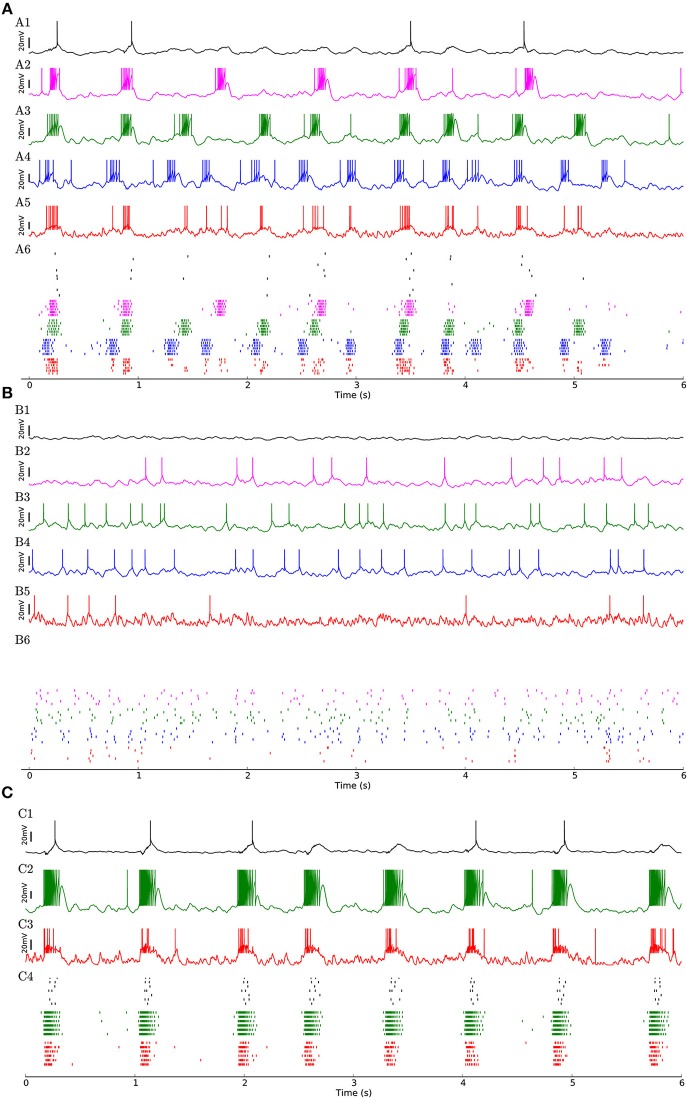
**(A)** Network of 454 excitatory and 90 inhibitory neurons with identical neuron parameters, organized into homogenous subpopulations with dense connectivity within each assembly and non-hubs. Membrane potential traces of a non-hub neuron **(A1)**, neurons from each of the three weight-hub assemblies **(A2, A3, A4)** and an inhibitory neuron (Inh) **(A5)**. **(A6)** Raster plot of several neurons of each population (same colors). Oscillations of the assemblies are different in terms of the up-state and down-state durations. Non-hub and inhibitory neurons receive input from the three oscillating assemblies and exhibit irregular oscillations. Note that there are 359 non-hub neurons in the network, which is the majority of cells. **(B)** Heterogeneous network as in Figure 2, but sparse connectivity (*p* = 20%) inside assemblies. Membrane potential of non-hub **(B1)**, three weight-hub neurons **(B2–B4)** and inhibitory neurons **(B5)** and the raster plot of several neurons of each population **(B6)**. The Up-state/down-state oscillation vanishes. Weight-hubs (**B2–B4**) occasionally emit spikes since they receive stronger synapses from Poisson neurons, while non-hub neurons **(B1)** do not spike at all. **(C)** Oscillations in a network with a single assembly of densely connected weight-hubs are more regular than in Figure 2. Membrane potential of a non-hub **(C1)**, a weight-hub **(C2)** and an inhibitory neuron **(C3)**. **(C4)** Raster plot of randomly selected neurons of each population. While weight-hub neurons (green ticks) exhibit a high firing rate in the up-state, non-hub neurons (black ticks) show only a small number of spikes. Inhibitory neurons (Inh) are shown in red.

To investigate whether the network structure, the broad distribution of synaptic weights, and the variation of neural parameters values are important for these dynamics, we built a similar model in which all synaptic weights inside a given subpopulation (i.e., weight-hubs, non-hubs, and inhibitory) are identical (See Section Materials and Methods) and values of neural parameters inside each subpopulation are identical (Table 1). The model still produces the irregular up/down state oscillations (Figure 3A).

On the other hand, the reduction of the connection probability inside the assemblies of weight-hub neurons from 50 to 20% (while maintaining the overall connection probability such that it agrees with experimental data) causes the dynamics to change significantly, and the irregular oscillations vanish (Figure 3B) even though network and neuronal parameters are heterogeneous. Thus, the connection probability inside weight-hub neurons assemblies plays an important role in the model dynamics. This is consistent with the model of Litwin-Kumar and Doiron (2012), in which clusters of neurons were predefined. Also in their study, decreasing the connection probability inside the neuronal clusters causes the transitions of clusters between active and inactive states to cease. In a related model (Mazzucato et al., 2015), in which inter- and intra-cluster connection probabilities are equal, decreasing the synaptic weights inside the clusters leads to a loss of oscillations.

Finally, we also simulate a network with a single assembly of densely connected weight-hubs (Figure 3C). While transitions between up and down states occur, the oscillations in non-hub neurons (and therefore the majority of neurons) are much more regular than in the full model of Figure 2 (the coefficient of variation of up-state duration for non-hub neurons in Figure 3C is 0.08, while it is 0.42 for Figure 2).

#### Simulation of optogenetic stimulation

Here, we explore the light-evoked response of our model of barrel cortex networks. Mimicking a recent experiment (Beltramo et al., 2013), a small subset of model neurons is stimulated to fire. Then, the activity of several non-stimulated neurons is recorded to investigate the relation and effect of the stimulated subset on the other neurons in the network. In our framework, an increased connection probability between weight-hub neurons can potentially explain the observation of the experiment: Optogenetic stimulation of a group of L5 neurons causes a long-lasting depolarization in non-stimulated L5 excitatory neurons, while the same experiment shows different results in L2/3 (Beltramo et al., 2013). Non-stimulated neurons in L2/3 show little depolarization and a smaller number of emitted spikes upon optogenetic stimulation of L2/3. These results imply that the L5 excitatory population is able to spread the optically induced activation more than the L2/3 one. In principle, this difference could be due to neuron parameters, neuronal morphology or the structure of neuronal networks. Here we argue that the presence or absence of densely connected weight-hubs assemblies, which is a property of the network structure, can explain the difference in the spread of activation within each layer. We propose that weight-hubs may be densely connected in L5, but their connectivity may be sparse in L2/3. This difference can be considered as one of several possible ways for interpretation of different light-evoked response in L2/3 and L5. Another noteworthy point is that although both connection probability and average synaptic weights in L2/3 are lower than L5 (see Section Materials and Methods), the number of neurons in L2/3 is much larger than L5 in our framework (1691 vs. 454). Therefore, the number and total strength of inward synapses into L2/3 neurons are not lower than L5 neurons.

**Figure 4:**
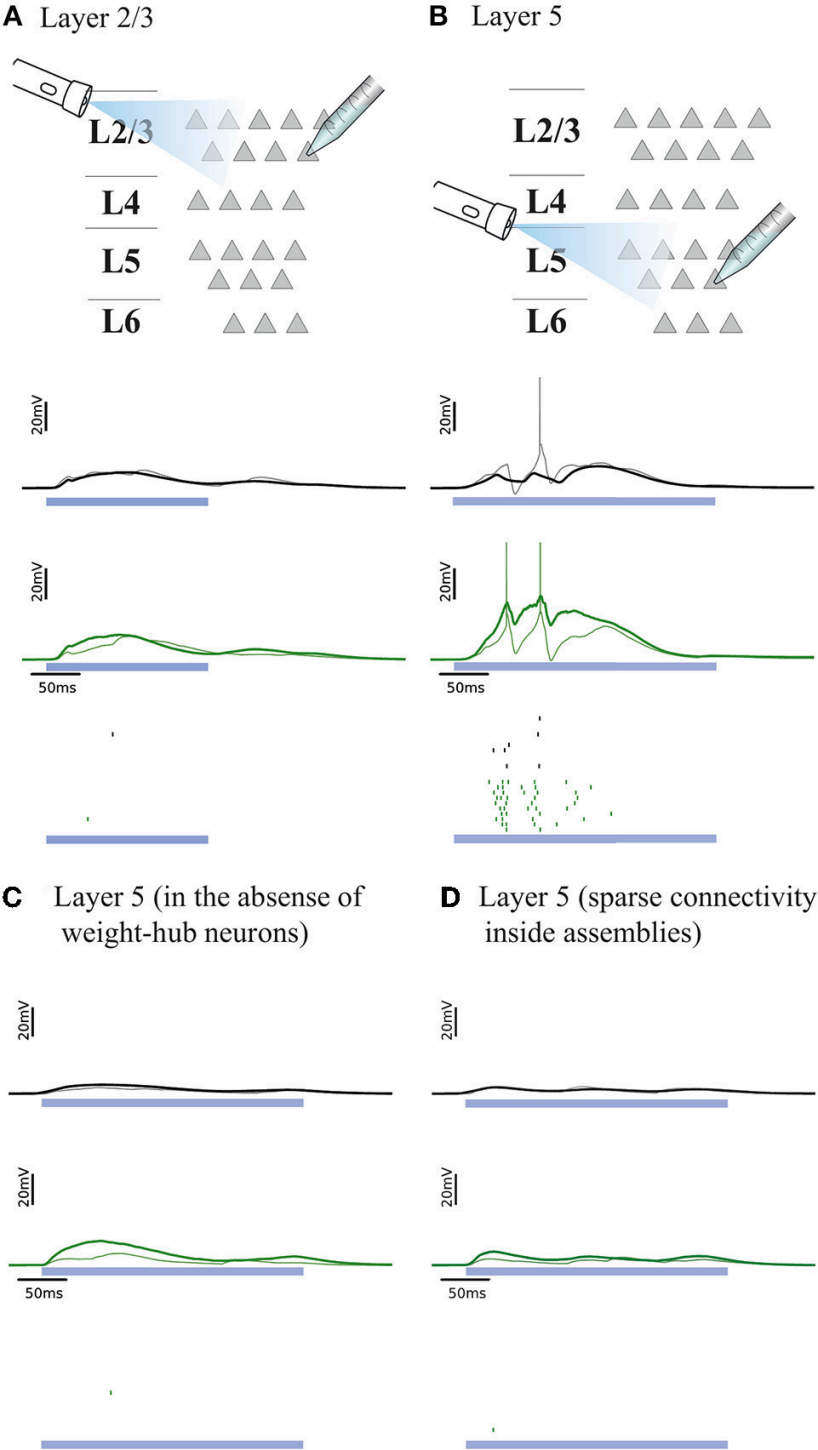
Simulated response to light-evoked stimulation of non-stimulated excitatory neurons in cortical L2/3 **(A)** and in L5 **(B)**. Approximately 15% of all neurons (weight-hubs, non-hubs and inhibitory) are stimulated in each layer for a time period of 300 ms (blue bar). Membrane potentials (lines) and spikes (ticks) of weight-hubs (green) and non-hubs (black). L2/3 neurons **(A)** show little depolarization due to sparse connectivity between weight-hubs, while L5 neurons **(B)** display a long-lasting depolarization and a significant number of spikes. This effect is due to the dense connectivity between weight-hubs in the L5 network model, but not in the L2/3 model. **(C)** Simulation of L5 in case of a modified assembly model that only has strong internal synaptic weights but is not innervated as strongly from other neurons as the weight-hub assembly in **(B)**, see main text for details. In the absence of weight-hub neurons, L5 does not generate long-lasting depolarization in response to the stimulation. **(D)** Simulation of L5 in case of having sparse connectivity (*p* = 20%) inside assemblies. The network is unable to produce long-lasting depolarization.

We examined whether the experiments in L2/3 and L5 can be explained by our cortical network model. Figures 4A,B, respectively, show the responses of L2/3 and L5 excitatory population models to a transient direct current stimulus, which we used to model optogenetic stimulation (Beltramo et al., 2013). The stimulus is received by a random subset (15%) of excitatory neurons, to account for the fact that about 15% of experimentally observed neurons express the light-sensitive ion channel. In the L2/3 population model, the connections within the weight-hubs assembly are sparse, whereas in the L5 model they are dense. Non-stimulated model neurons in L2/3 show little depolarization while L5 ones show a high depolarization and a substantial number of spikes, in agreement with experiments in mouse visual cortex (Beltramo et al., 2013). Therefore, we conclude that dense connectivity between weight-hub neurons in L5 and sparse weight-hub connectivity in L2/3 can generate biologically plausible light-evoked responses.

In the simulations of Figures 2, 3, we observed that densely connected weight-hubs produce up-state/down-state oscillations while sparsely connected ones do not. In the simulation of Figure 4, we set up L5 to contain densely connected weight-hubs and L2/3 to contain sparsely connected ones. Therefore, in this model L5 is the main source of the up/down oscillations in the cortex, while L2/3 is subsidiary. Other experimental studies (Sakata and Harris, 2009; Chauvette et al., 2010; Beltramo et al., 2013) provide further support of this conclusion.

To assess whether the concept of weight-hubs is necessary to explain the light-evoked responses, we modified the structure of L5 network and repeated the simulation. In the new structure, we did not use weight-hub neurons (neurons that receive strong synaptic weights from all other neurons) but we instead implemented an assembly with high connection probability and strong synaptic weights internally. In other words, in the new structure the neurons of the assembly receive strong synaptic weights from each other while they receive normal weights (broadly distributed weak and strong weights, as shown in Figure 1B) from the neurons outside the assembly. Our simulation shows that this structure is unable to produce a long-lasting depolarization and a notable number of spikes (Figure 4C), indicating that the weight-hub property is important to explain the light-evoked response of L5. We also simulated light-evoked response of another variant of L5 model (Figure 4D). We showed that L5 in presence of sparsely connected weight-hub neurons cannot generate long-lasting depolarization.

#### Correlations

**Figure 5:**
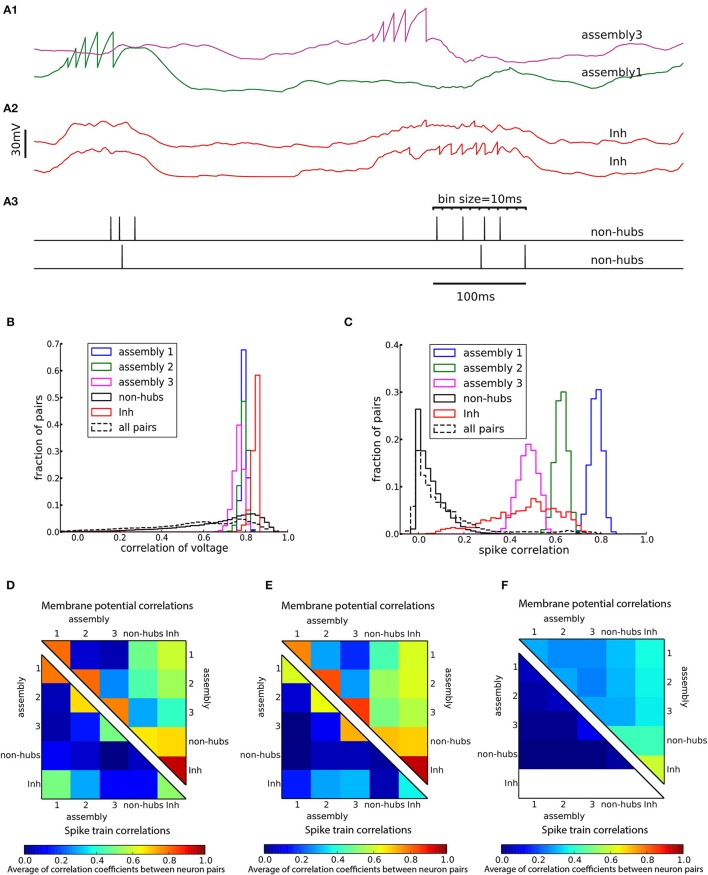
Cross-correlations of neuronal activity. **(A)** Two pairs of subthreshold membrane potentials (spikes have been removed) with low **(A1)** and high **(A2)** correlation and a pair of spike trains **(A3)**. Spikes are counted as coincident if they fall within the same bin of 10 ms. **(B,C)** Distribution of Pearson correlation coefficients of subthreshold membrane potentials **(B)** and spike trains **(C)** of pairs of neurons inside each subpopulation (solid lines) and over all 145,530 pairs of neurons (dashed lines). **(D–F)** Averaged Pearson correlation coefficients between the membrane potentials (upper triangle) and the spike trains (lower triangle). Correlations are computed for pairs of neurons in the respective subpopulations of **(A–D)** the heterogeneous network of Figure 2, **(E)** the homogeneous network of Figure 3A and **(F)** the sparsely connected weight-hubs network of Figure 3B. Because inhibitory neurons do not fire any spikes in the sparsely connected weight-hubs network **(F)**, the spike train correlations of them are not defined (white area).

A characteristic of weight-hub assemblies in the model (Figure 2) is that the activity of hub neurons is strongly correlated. We quantified correlations by computing the correlation coefficients of pairs of neurons, both of the subthreshold membrane potentials and the spike trains, binned into a 10 ms time window (Figure 5A). In the heterogeneous network of Figure 2 the subthreshold membrane potentials of neurons inside each subpopulation are strongly correlated (Figures 5B,D; mean correlation coefficient 0.80, 0.79, 0.75 for assemblies 1, 2, and 3, respectively, and 0.94 for inhibitory neurons) and significantly smaller (Student's *t*-test for difference in mean: all *p*-values are smaller than 10^−10^) for non-hubs (mean correlation coefficient 0.65). In contrast, correlations between neurons of different weight-hub assemblies are small, because their oscillations are not synchronized (Figure 5D). The correlation analysis of the spikes generated in the heterogenous network of Figure 2 also shows correlated behavior inside subpopulations except for non-hubs (Figures 5C,D). The mean correlation coefficients for the spike trains of the non-hubs is 0.06, smaller (Student's *t*-test for difference in mean: all *p*-values are smaller than 10^−10^) than that of other subpopulations (mean correlation coefficient 0.79, 0.65, 0.42 for assemblies and 0.52 for inhibitory neurons). If we randomly select two neurons in the network, we find a broad distribution of pairwise spike correlations (Figure 5C, dashed line) with a peak close to zero, consistent with experimental data (Reich et al., 2001) and previous model of metastable dynamics (Mazzucato et al., 2016). Heterogeneous (Figure 5D) and homogeneous (Figure 5E) variants of the model show very similar correlation structure, but in the network with sparsely connected weight-hub neurons (Figure 5F), the correlations disappear, because assemblies are mainly driven by external noise and do not show any joint transitions to the up-state (Figure 3B).

#### Active cortical state

**Figure 6:**
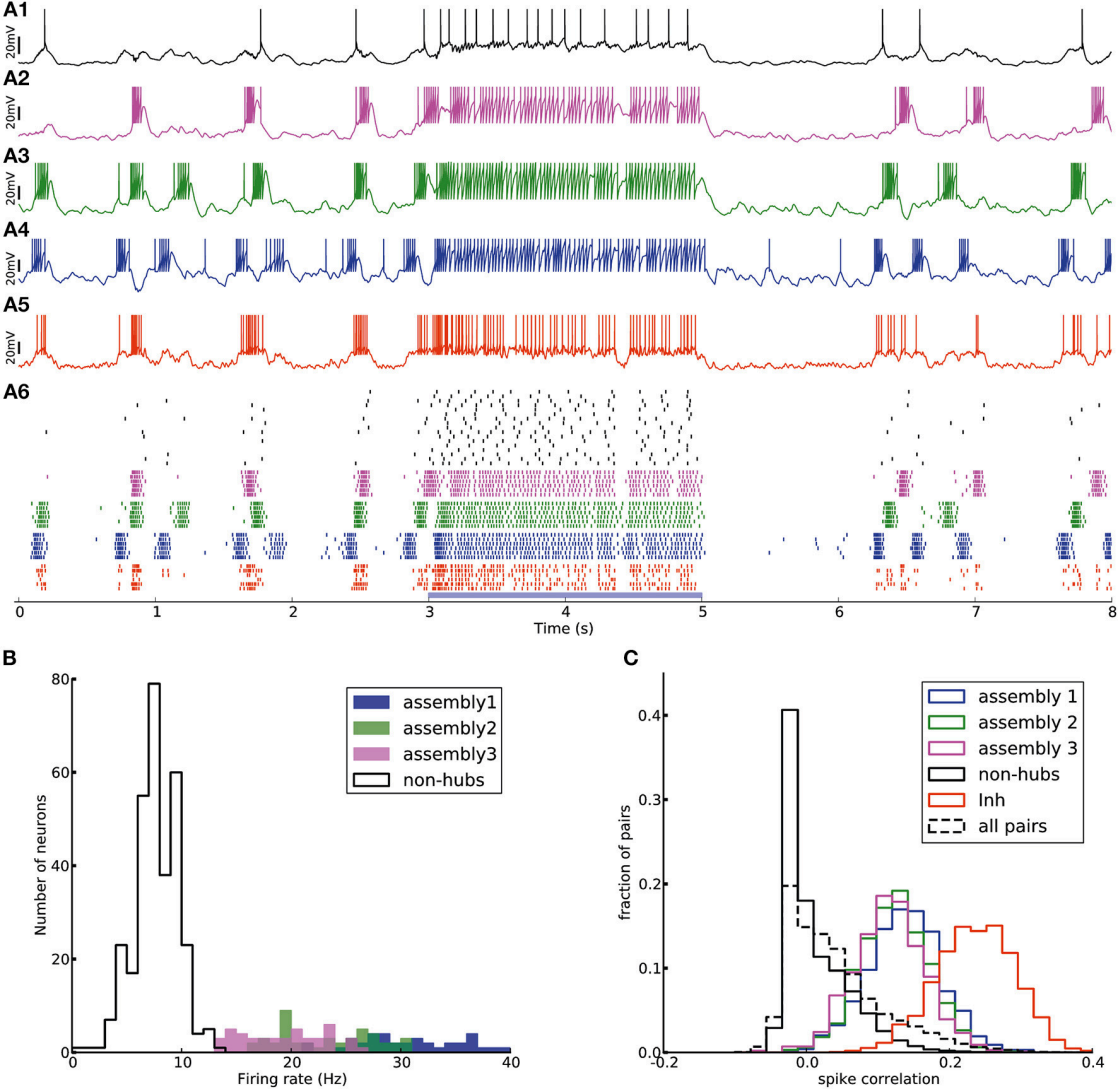
Transition from up-down-state oscillations to “active” state. **(A1–A6)** Network of Figure 2 receiving external Poisson process stimulus from *t* = 3 s to *t* = 5 s (blue bar). Neurons show up-down state oscillation before and after the stimulus, while they exhibit higher firing rate (20.9 Hz for inhibitory neurons and 12.4 Hz for excitatory neurons, split into 37.4, 33.6, 33.0, 6.4 Hz for assemblies 1, 2, 3 and non-hubs neurons respectively) during the stimulation period. **(B)** Distribution of firing rates across neurons in the network during stimulation interval (blue bar in **A**). **(C)** Distribution of Pearson pairwise correlation coefficients (bin size = 10 ms) of spike trains of pairs of neurons inside each subpopulation (solid lines) and over all pairs of neurons (dashed lines) during stimulation interval.

Our network model can switch from the oscillatory state (which resembles slow-wave sleep or anesthesia) to an active state and vice versa without any change of network properties. In particular, the assemblies of weight-hub neurons, which are responsible for producing the up-down state oscillations, are always embedded in the network. In the active state, cortical neurons receive sensory input predominantly from layer 4 and layer 6 neurons which relay the sensory signals between thalamus and other cortical layers (Binzegger et al., 2004; Poulet et al., 2012). Here we simulate the active state by injecting an external stimulus (homogenous Poisson process to generate excitatory spike trains) to all neurons of the model. Figure 6A shows that the network stops oscillating immediately after receiving the stimulus, and switches back to the up-down state oscillations when the stimulation stops.

The effect of the external input can be explained as follows. When there is no strong external input, the network is driven by the dynamics of the weight-hub assemblies. Since they show self-sustained oscillations, the whole network is affected by the oscillations and follows them. In case the external input is present, however, the network is driven mainly by the input (even if the input is stationary, as in the case driving our stimulus with a homogenous Poisson process) and not the assembly dynamics. Therefore, all neurons, including the weight-hub neuron assemblies, are governed by the input and stop oscillating. During the input-driven active state, the firing rate distribution is narrow (Figure 6B) and the correlation between neurons is very low (Figure 6C). The average correlation coefficients for the active state (0.15, 0.14, 0.12 for assemblies 1, 2, and 3, respectively, and 0.25 for inhibitory neurons) are smaller than in the oscillatory state (Figure 5C; Student's *t*-test for difference in mean: all *p*-values are smaller than 10^−10^). The very low average correlations between (over all neuron pairs, 0.04 ± 0.05, Figure 6C, dashed line) and inside excitatory neurons (Figure 6C, black line) are consistent with recent experimental observations (Ecker et al., 2010). Therefore, the network preserves important aspects of biologically plausibility (such as skewed firing rate distribution and low value of pairwise correlations) also in the active cortical state.

### The role of the weight-hub neurons assembly in the slow oscillations

**Figure 7:**
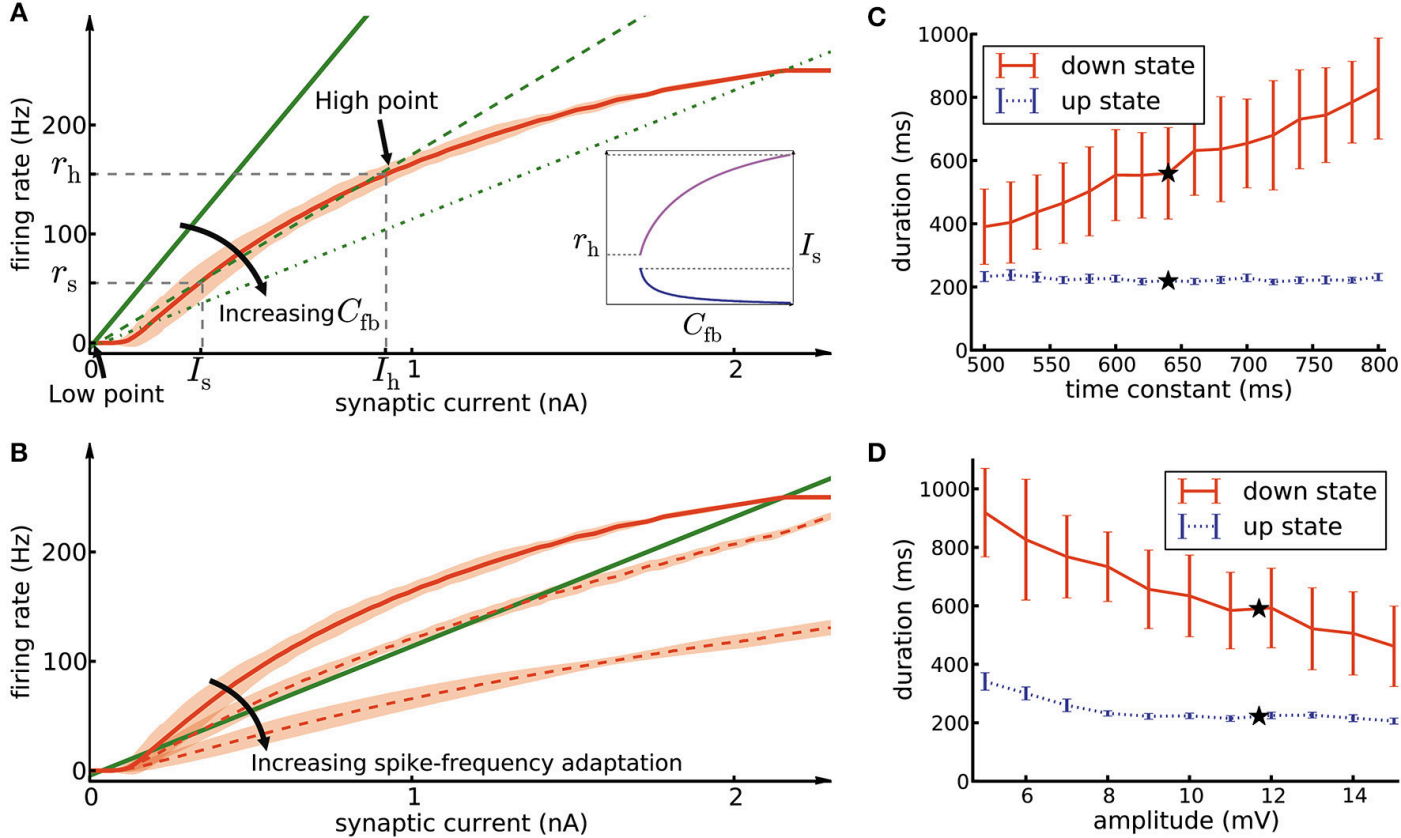
Mean-field analysis. **(A)** The network feedback (*C*_fb_, Equation 17) affects the quasi-stationary dynamics of the system. The red curve is the noisy gain function *g* of the GIF neuron model (mean spike count in a group of 50 independent neurons over 10 ms, divided by 50 × 10 ms, shaded area marks ±3 SEM) measured during the initial 10ms after switching on a synaptic current of mean 〈*I*_syn_〉 (see Section Materials and Methods, Equation 14). The green lines (solid, dashed, and dash-dotted) show the relation of firing rate and synaptic current caused by network feedback (see Section Materials and Methods, Equation 16) for increasing *C*_fb_. The slope of the green lines has an inverse relation with the effective coefficient *C*_fb_ of the population. Intersections of the red curve with one of the green lines indicate potential stationary states (fixed points) of a network of non-adapting neurons. Populations with a high *C*_fb_ (dashed and dash-dotted green lines) have three fixed points, stable low point, high point and unstable switch point. If the population described by a network feedback given by the dashed lines is driven by a mean current higher than *I*_s_, it rapidly converges to the high point. On the other hand, a population with a low *C*_fb_ (solid green line) has only one intersection at the low point. Inset: Increasing *C*_fb_ causes an increase in the high firing rate *r*_h_ (magenta curve, left vertical scale) and a decrease of the switch current *I*_s_ (blue curve, right vertical scale). **(B)** The noisy gain function of adaptive neurons is different during the first 10 ms after stimulus onset (solid red curve) than later (dashed red curves) **(C)** The duration of up- and down-states as a function of the time constant of the excitatory neuron firing threshold kernel γ(*s*). Only the time constant of γ_2_(*s*) (the exponential with the longer time constant) was manipulated, while γ_1_(*s*) remained as reported in Table 1. The black stars indicate the experimentally extracted value of the time constant, which was used in the other figures. **(D)** Same as C, except that here we manipulated the amplitude of γ_1_(*s*) (the exponential with the larger amplitude). The error bars show the standard deviation of up/down state durations over 10 simulation trials of 10 s duration each.

In order to understand why the assembly of densely connected weight-hub neurons generates oscillations, we use methods from network analysis (Amit and Brunel, 1997; Laing and Chow, 2002; Giugliano et al., 2004, 2008; Moreno-Bote et al., 2007; Shpiro et al., 2009; Gerstner et al., 2014; Mazzucato et al., 2015). We relate the mean firing rate of neurons of the assembly to the mean synaptic current received by them. The first relation is given by the neuronal gain function (Equation 13, curve in Figure 7A), i.e., the firing rate that each neuron produces when it is driven by a certain input current. The second relation is given by the feedback of the network (Equation 16, lines in Figure 7A), i.e., how much synaptic current is produced by the activity of the neurons. In the absence of adaptation, intersection points between the two curves form candidates of fixed points of network activity. We define the “network feedback” (*C*_fb_, Equation 17) as the strength of synaptic feedback within the assembly. This quantity is the inverse of the slope of the feedback line (lines in Figure 7A). A high (low) value of *C*_fb_ leads to a strong (weak) response of the assembly to synaptic currents.

In the framework of up- and down-state oscillations, an assembly (or any subpopulation of neurons) with low value of *C*_fb_ is not able to oscillate and remains in a low firing rate fixed point. This can be explained as follows: A low value of *C*_fb_ implies that the feedback line has a large slope (solid line in Figure 7A). Therefore, the two curves have only one intersection point whose rate is close to zero. We refer to this fixed point as “low-point.” In the case of a high value of *C*_fb_, the slope of the feedback line is low (dashed lines in Figure 7A) and there are two additional fixed points of the system. The middle fixed point is unstable and is called the “switch point,” and the upper one is typically stable (neglecting adaptation and oscillatory instabilities; Brunel, 2000; Gerstner, 2000) and is called the “high point.” Let us refer to their positions with symbols (*I*_s_, *r*_s_) and (*I*_h_, *r*_h_) with indices *s* and *h* for “switch” and “high,” respectively. *I*_s_ acts as a threshold for the behavior of the assembly. In case of driving neurons with a current lower than *I*_s_, the assembly converges to the low-point and remains quiescent. In contrast, currents higher than *I*s bring the assembly to the high-point and force it to exhibit a high firing rate. In our framework external Poisson noise occasionally provides a transient synaptic current larger than the current of the switch point. The mechanism described above is the reason that the assembly switches from the low-point to high-point. Switching back from the high-point to the low-point is due to spike-frequency adaptation and will be discussed further below.

As discussed, a sufficiently large value of *C*_fb_ gives rise to two stable fixed points of the network activity, the low-point and the high-point. Since the assembly of connected weight-hub neurons exhibits high average synaptic weights (*w*_h_) and high connection probability (*p*_h_), the value of *C*_fb_ ∝ *w*_h_*p*_h_ for this subpopulation is high (see Section Materials and Methods, Equation 17). But is such an assembly of weight-hub neurons necessary for producing the oscillations? Or can any highly connected group of neurons (not necessarily weight-hub neurons) generate the oscillation? Or even a group of neurons with very strong synaptic weights but sparse connectivity (similar to Mazzucato et al., 2015)? The relevant parameters for the configuration of fixed points is the value of *C*_fb_, which can in principle be increased by an increase of either *w*_h_ or *p*_h_, or both. However, we found some of these possibilities to be not consistent with the existing experimental data. In particular, fixing *p*_h_ and increasing *w*_h_ by a large factor yields a set of very strong synapses out of the range of reported experimental PSP values (Lefort et al., 2009). On the other hand, by fixing *w*_h_ we require a very high *p*_h_ (close to full connectivity, *p*_h_ = 1) which does not look biologically plausible for cortical networks (for more details of relation between connection probability and other population parameters see (Klinshov et al., 2014)). For example, in the case of the smallest assembly (containing 20 neurons), choosing sparse connectivity *p*_h_ = 0.2 yields to very high synaptic weight value *w*_h_ = 3.55 mV, similarly choosing synaptic weight *w*_h_ = 0.71 mV (average synaptic weight in L5 excitatory population is 0.66 mV) leads to full connectivity (*p*_h_ = 1). Our solution was to increase both *w*_h_ and *p*_h_ by a moderate factor, so that both remain realistic and still lead to a sufficiently high value of *C*_fb_.

We would like to highlight another characteristic of *C*_fb_. Increasing its value increases the firing rate *r*_h_ in the high fixed point, and lowers the minimal value of the switching current *I*_s_ (Figure 7A, inset). Consequently, a high value of *C*_fb_ implies that only a small amount of transient external current is required to bring the population above the switch point. In our model, different assemblies have different numbers of neurons, therefore different values of *C*_fb_, and different switching points. The different feedback coefficients of weight-hubs assemblies and non-hub neurons also explain the skewed distribution of firing rates (Figure 2B) in the model. Larger assemblies of weight-hub neurons switch more often to the high-point and produce higher firing rates than smaller assemblies. Hence, weight-hub neurons from different assemblies form the tail of firing rate distribution. Non-hub neurons do not switch to the high-point and are not able to produce high firing rate. Therefore, they form the peak of the firing rate distribution at low rates.

Let us now focus on the return of the assembly from the high-point to the low-point. At the high-point neurons exhibit a high firing rate but spike-frequency adaptation continuously decreases the probability of spike emission. Therefore, the neurons' gain function changes gradually (dashed curves in Figure 7B). The system eventually makes the transition to a new configuration where the low-point is the only fixed point. When the system is at the low-point, both adaptation current and dynamic threshold decay, and eventually the dynamics of the subpopulation go back to the initial configuration in which both high- and low-point exist. In other words, the neurons recover from adaptation while they are in the low-point. Stochastic spike arrivals from other areas, described as Poisson neurons with constant firing rate here, provide the excitation necessary to make the assembly switch to the high-point. The resulting process of repetitive switching between the low-point and the high-point forms the oscillation in the system. The high point, corresponding to high firing rates of the weight-hub neurons in our model, can be interpreted as the up-state of a cortical network, and similarly the low-point corresponding to low firing rates of the down-state. Previous studies (Giugliano et al., 2004, 2008) addressed these dynamics for a simpler adaptive integrate-and-fire neuron model with similar analytical approaches.

Because spike-frequency adaptation in our model is responsible for progressively changing the gain function during the up-state, and eventually for its termination, we investigate the effects of the adaptation parameters on the duration of the up- and down states. Each time a neuron emits a spike, several adaptation processes are added to its firing threshold and spike-triggered current (denoted as “kernels” γ(*t*) and η(*t*) in Table 1). Each kernel has an exponential form of *be*^−*t*/τ^, where *b* and *t* are amplitude and time constant of the kernel, respectively, and t is the time elapsed since the emission of the spike. By modifying the time constant *t* of the firing-threshold kernel γ_2_(*t*) (which has a longer time constant than γ_1_(*t*)) for the excitatory neurons, we are able to change the down-state duration strongly without affecting the up-state duration (Figure 7C). A longer time constant implies that neurons need more time to recover from adaptation, which leads to a longer duration of the down-state. On the other hand, manipulation of the kernel amplitude affects both up- and down-state durations, as shown by changing the amplitude of the exponential term of the kernel γ_1_(*t*) (Figure 7D). Longer values of the amplitude cause shorter up- and down-states. Note that because the switch from the down-state to the up-state in our model is caused by stochastic Poisson inputs, the down-state durations have a greater variability compared to the up-state durations (see error bars in Figures 7C,D). In general one could manipulate amplitudes and time constants of all exponential kernels in γ(*t*) and η(*t*) as well as other neuronal parameters. However, since all neural parameters of our model (including the adaptation parameters) are extracted from experiments (Mensi et al., 2012), we did not investigate manipulating them any further.

In order to examine whether the above network feedback mechanism is indeed causing the up-down state transitions, we simplified the model of L5 such that it contains a single assembly of densely connected weight-hubs (see Section Materials and Methods) embedded in a network of non-hubs with weak random connections. Figure 3C shows the dynamics of the network. Note that in this model the assembly acts as the driving force of the system and generates an oscillation by switching between its two stable fixed points. The non-hub neurons are enslaved by this oscillation and show only the passive behavior of followers. However, while embedding only one assembly in the excitatory population makes the system oscillate, the duration of up-states is short and has a narrow distribution (regular duration). In contrast, a combination of several assemblies, as in Figures 2, 3A, results in several oscillations with different frequencies, each of which is generated by one assembly. Non-hub and inhibitory neurons receive these excitatory input signals and superimpose them. The result of the superposition are rather irregular up-states with a longer duration (Figures 2, 3A). The role of inhibitory neurons in the model is to regulate the firing rate of the assemblies in the up-state. This regulation is necessary because an excessively high firing rate in each assembly would cause rapid spike-frequency adaptation, and would therefore substantially reduce the duration of the up-states in that assembly. Consequently, the superimposed oscillations would also show short up-state durations.

We can also explain the light-evoked response of L5 and L2/3 (Figure 4) by the dynamics of the assemblies of weight-hub neurons. In order to understand the differences between the layers, let us suppose that most of the neurons that express the light-sensitive ion channel generate one or several spikes in response to the stimulus. Assuming a uniform spatial distribution of weight-hubs in the excitatory population, we estimate that ~15% of both weight-hub and non-hub neurons are stimulated. Since synaptic weights among non-hubs are weak, non-hub neurons do not strongly excite neighboring non-hub neurons. However, since they send strong synapses to weight-hubs (according to our definition of weight-hubs), they contribute to the initial activation of weight-hub neurons. Recall that a densely connected assembly needs only little initial activation (to reach the switch point) to generate a high firing rate via self-excitation. Therefore, a densely connected assembly switches to the high point more easily so that each weight-hub neuron fires several spikes.

In contrast, since the connections from weight-hub neurons to non-hub neurons are weak, the stimulation does not generate a high overall firing rate in the network (non-hub neurons depolarize but do not show a high firing rate). After the weight-hub neurons have fired several spikes, spike-frequency adaptation changes the neuronal gain function. This switches the mean-field dynamics of the weight-hub assembly from a three-fixed-point to a one-fixed-point regime and brings the assembly to the low-point (Figure 7B), as discussed above. Consequently, both firing in weight-hubs and depolarization in non-hubs cease. In contrast, in the case of sparse connectivity between weight-hub neurons, due to a low value of the *C*_fb_, this subpopulation is unlikely to transition to the high-point. Either such a fixed point does not exists because the system has only one fixed point (the low-point), or the value of the switch current (*I*_s_) is very high and stimulated neurons cannot provide sufficient input current to reach it. Therefore, the absence of a densely connected weight-hub assembly leads to weak spreading of the induced activation in the population (Figure 4B). For the case of Figure 4C, since synaptic weight from non-hub neurons onto assembly neurons are weakened, assembly neurons do not receive enough synaptic current to cross the switch current. Consequently, they cannot produce notable number of spikes.

## Discussion

In this paper, we suggest cortical microcircuits with a particular non-random network feature called assembly of densely connected weight-hub neurons, to explain two different experimental observations: Firstly, spontaneous slow oscillations (irregular up- and down-state) and, secondly, stimulus-evoked responses of cortical layers.

We argue that in our framework the existence of weight-hub neurons in a cortical network alone is not enough to cause significant changes in network dynamics. Since, we want the values of network parameters (synaptic weights and connection probabilities) to remain in the experimentally observed range, we may not increase the synaptic weight of connections on weight-hub neurons by a huge factor. Therefore, the value of network feedback cannot become high enough to produce oscillations by only modifying the weights. But if the connection probability between weight-hubs is also high (at least twice the connection probability between two arbitrary non-hub neurons), the emerging assembly of densely connected weight-hubs shapes the dynamics and the activity of the cortical layer. We have shown both qualitatively and quantitatively that a small but sufficient amount of initial activation brings the assembly of model neurons to a transient high-rate state that resembles cortical up-states.

A single assembly of weight-hub neurons together with a small amount of external noise (which here is provided by constant rate Poisson inputs) forms a slow oscillator. The reason is that this assembly switches between a high-rate state and a low-rate state repetitively. Fluctuations caused by external noise bring the assembly to the high-rate state, and spike-frequency adaptation brings it back to the near-zero, low-rate state. Several experimental studies (Sanchez-Vives and McCormick, 2000; Sakata and Harris, 2009; Chauvette et al., 2010; Beltramo et al., 2013) indicate that the cortical oscillations originate in infra-granular layers (mainly L5), and that supra-granular layers (L2/3) are subsidiary, i.e., the up-state is initiated in L5 and rises from the depth to L2/3. We suggest that the connectivity of weight-hub neurons in L5 is dense, while it is sparse in L2/3. Thus, L2/3 follows oscillations generated in L5, but is not able to sustain oscillations on its own.

In slice cultures slow oscillations are rather regular (Sanchez-Vives and McCormick, 2000), whereas experiments done in the anesthetized animals (Stern et al., 1997; Lampl et al., 1999) show irregular up-down state transitions. In order to reproduce this irregularity, we embedded several densely connected weight-hub assemblies in the excitatory population. Non-hubs, the majority of excitatory cells, and inhibitory neurons receive synaptic input from these oscillations and superimpose them. Consequently a large fraction of model neurons show an irregular oscillation with a broad distribution of up-state durations.

The presence of one or several assemblies of weight-hub neurons may also explain layer-dependent differences of stimulus-evoked responses (Beltramo et al., 2013). While L2/3 exhibits weak depolarization in response to stimulation of a small fraction of it, in L5 the same stimulus induces a strong and long-lasting depolarization and a substantial number of spikes. Since the assembly of weight-hub neurons needs just a small amount of activation to switch to a high firing rate, and may propagate it within the network, we suggest that the connectivity of weight-hub neurons underlies the long-lasting response of L5. Conversely, we would hypothesize that weight-hub neurons in L2/3 are not strongly connected to each other.

While our multi-assembly architecture produces long tailed distribution of firing rates, there is at least one other way to produce such a skewed distribution. In a balanced network in the asynchronous state (Roxin et al., 2011) showed that a Gaussian input distribution can lead to a lognormal firing rate distribution via an exponential non-linearity of the current-frequency relation. In our network model, however, neurons are not in the balanced stationary regime but participate in synchronous transitions between up and down states. Similarly, in the study of Mazzucato et al. (2015) the long tailed distribution of firing rates results from metastable activity not from the balanced stationary state.

### Definition of hub neuron

The term hub can have two meanings: Firstly, degree-hub, i.e., a neuron that receives more synaptic connections than an average neuron, and secondly, weight-hub, i.e., a neuron that receives stronger synapses than average. The second definition was used in the current work. The common definition of a hub (degree-hub) as a neuron that receives more synapses (Bullmore and Sporns, 2009; Feldt et al., 2011; Prettejohn et al., 2011) does not take into account the strength of synapses, called synaptic weights here. This topological definition of hubs is common in computer sciences, where the issue of degree and connections between nodes is more important than the weight structure. However, in neuronal microcircuit modeling, synaptic weights are as important as degree and connectivity. Surprisingly, the amount of previous modeling work done on degree manipulation and connectivity structure (Roxin, 2011; Hu et al., 2013, 2014; Pernice et al., 2013; Vasquez et al., 2013; Jahnke et al., 2014; Potjans and Diesmann, 2014; Rudolph-Lilith and Muller, 2014) by far exceeds work on non-homogeneous weight structure (Koulakov et al., 2009; Iyer et al., 2013; Tomm et al., 2014).

Here we adopted the less-common definition of a hub in terms of synaptic weights, to shed light on this less-well understood aspect of non-random neuronal network features. While manipulation of the degree distribution in the network and creating degree-hubs has the same effect as creating weight-hubs in producing a skewed firing rate distribution (Roxin, 2011), the two are not always interchangeable. Tomm et al. (2014) showed that for reproducing light-evoked responses in mouse barrel cortex slices, manipulation of both degree and weight distributions are needed under the simulated network conditions of this study. In our case, it would be possible to keep the connectivity random (without rewiring) and manipulate the weights more strongly to produce the oscillations. However, this would entail very strong synaptic weights outside of the experimentally observed range (Lefort et al., 2009), and thus would reduce the biological plausibility of the model. Our combined approach of introducing weight-hubs with dense connectivity overcomes this problem with minimal changes to both weight and degree distribution.

Several electrophysiological experiments indicate that the distribution of synaptic weights (EPSP amplitudes) has a lognormal shape (Lefort et al., 2009; Ko et al., 2011; Avermann et al., 2012; Chapeton et al., 2012). Therefore, we used a lognormal distribution for modeling synaptic weights in neuronal populations. Using random (Erdős–Rényi) networks to define whether a synaptic connection is present or not, together with a lognormal distribution for the synaptic weight of the connection, entails that the sum of inward synaptic weights is similar for all neurons (Figure 1B). However, by modifying the topology of the excitatory network to have local inward correlation in synaptic weights (see Section Materials and Methods), we could produce a more broadly distributed sum of inward synaptic weights (Figure 1B). Therefore, a few excitatory neurons receive larger total synaptic weights, while others receive smaller values. For the sake of concreteness, we have defined a classification boundary such that the 20% of neurons that receive the strongest inputs are called weight-hubs (see Figure 1B). The model is robust against varying this fraction, and shows similar dynamics for 15 and 25% of neurons (data not shown).

Throughout this article, only excitatory to excitatory connections were manipulated and all other connections (inhibitory to inhibitory, inhibitory to excitatory and excitatory to inhibitory) remained unchanged. However, evidence of hubs in inhibitory populations has been found in experiments (Bonifazi et al., 2009). Therefore, the effects of hubs in inhibitory networks remain to be investigated. Since in our model excitatory hubs suffice to explain the aforementioned aspects of cortical dynamics, we neglected inhibitory hubs here in favor of model simplicity. Another reason for not exploring the structure of inhibitory connections is the lack of experimental datasets for inhibitory connectivity in L5. In this work, we use the inhibitory connection properties of L2/3 as a substitute for L5 (see Section Materials and Methods). Therefore, any investigation on inhibitory connectivity of L5 would be based on this unconfirmed hypothesis.

### Identifying weight-hub neurons from data

A network structure with densely connected weight-hubs is hypothetical. In this section, we propose a method using machine learning tools which can help experimentalists to label neurons as weight-hub or non-hub using a set of recorded membrane potential. In order to perform such a classification, we need to consider distinct properties of weight-hub neurons. First, weight-hub neurons in our model receive a larger amount of excitation than non-hubs and therefore exhibit a higher firing rate. Therefore, we might label neurons with relatively higher firing rate as weight-hubs. However, this property alone does not yield a robust way of identification, because besides the synaptic input, the firing rate of a neuron also depends on its electrophysiological parameters, such as its firing threshold. A non-hub neuron may therefore occasionally exhibit a higher firing rate than a weight-hub (Figure 2B). A second property of weight-hub neurons predicted in the context of our model is the regularity of transitions between up- and down-states. According to our model, regular up-state durations indicate that the neuron is a weight-hub.

**Figure 8:**
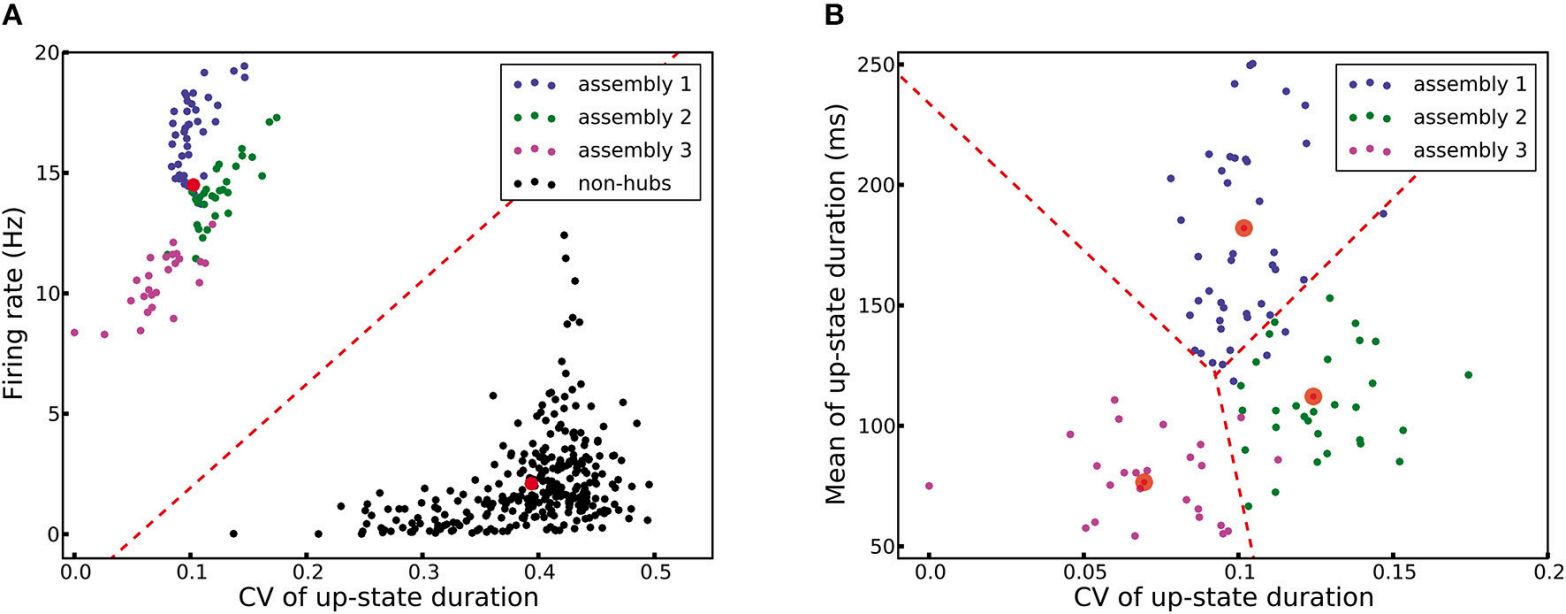
K-means clustering identifies weight-hub neuron assemblies. Each dot represents one neuron and its color denotes the corresponding subpopulation in the simulation shown in Figure 2. **(A)** Clustering of all neurons into two clusters. The first stage of the classification algorithm successfully identifies weight-hubs and non-hubs, but does not distinguish between different assemblies of weight-hubs. The red circles show the center of the clusters and the dashed line displays the classification boundary. **(B)** Clustering of weight-hub neurons (identified in **A**) into different assemblies. The K-means algorithm with three clusters identifies the assembly of each weight-hub neuron with 93.7% accuracy.

Here we use a combined approach for identifying subpopulations of weight-hub and non-hub neurons. In the first step, we characterize each neuron by a vector of two elements: the overall firing rate and the CV of up-state durations of the neuron. We pass these vectors to a K-means clustering algorithm (see Section Materials and Methods), which clusters neurons into weight-hubs and non-hubs with 100% accuracy (Figure 8A). We identify the group with the lower coefficient of variation of up-state duration as the weight-hubs and other group as the non-hubs. In order to distinguish the assembly that each identified weight-hub neuron belongs to, we perform a second step and run the algorithm again on the weight-hub neurons found in the previous step. Here we define the feature vector of each neuron by the mean and the CV of up-state durations. The algorithm assigns the correct assembly to 89 out of 95 weight-hub neurons. Accordingly the accuracy of the second step is 93.7% (Figure 8B).

Although our approach works for identifying of weight-hub neurons in our simulations, finding these neurons in the cortex using intracellular recording will be more challenging. This is mainly due to the fact that weight-hub neurons are likely to form only a small portion of all excitatory neurons. In our simulations of L5 of a single column in the barrel cortex ~20% of all excitatory neurons are weight-hubs. However, we can scale our model system up keeping the number of weight-hub neurons fixed, without a change to the overall dynamics. Because weight-hub neurons are generators of the oscillations and non-hub and inhibitory neurons follow them. We can increase the number of followers with the same number of weight-hub neurons. For example a similar model of L5 which contains both Layer 5A and Layer 5B (containing ~1,000 excitatory neurons) needs the same number of weight-hubs to display up-down state oscillations. Therefore, in this example, the fraction of weight-hubs reduces to 10%. Analogously, in case of modeling an entire barrel column (containing ~5,700 excitatory neurons), this number falls to about 1.7%. Therefore, we expect that weight-hub neurons are rarely recorded with present-day single-cell electrophysiological techniques.

### Different models for reproducing up/down oscillations

Several models have been suggested to reproduce up-down state oscillations. The studies of Ghorbani et al. (2012) and Holcman and Tsodyks (2006) used mean-field analysis to show that short-term depression can give rise to the up/down state oscillation. Other works focused on numerical simulations on the neuronal level: Applying short-term facilitation on excitatory to inhibitory connections also produces oscillations (Melamed et al., 2008), as well as adding a non-linear term to the leaky integrate-and-fire model such that each neuron is bistable (Parga and Abbott, 2007). Giugliano et al. (2004) used a homogenous network of adaptive neurons to produce oscillations using a similar mechanism as in our model. However, in their model all the neurons switch between the high- and low-rate points. Hence they all have a high firing rate, and the distribution of firing rates is less skewed. On the other hand, if we used the average connection probability and synaptic weights obtained by the experiments (Lefort et al., 2009) for building a homogeneous network with plausible size, the value of the network feedback would not be high enough to make the network oscillate. Therefore, we consider the proposed network with embedded weight-hub neuron assemblies to be the most plausible model with respect to these data.

Switching between up- and down-states. In slowly oscillating cortical microcircuits, the reason for the switch from the down-state to the up-state is a matter of debate (Chauvette et al., 2010). One possible reason could be the coincidence of spontaneous activity of several neurons (Timofeev et al., 2000; Bazhenov et al., 2002). Such a coincidence may provide enough input to several other neurons to make them fire. This phenomenon then repeats and propagates the activity to a notable number of neurons, and so the system may switch to the up-state. Another possibility is that a few neurons are active more than others on average, and show firing even in the down-state. One hypothesis about such neurons is that they receive a persistent sodium current which causes bursts of firing (Hill and Tononi, 2005; Bon-Jego and Yuste, 2007). Another hypothesis is that electrophysiological properties of these neurons make them fire more than others, e.g., they can have lower firing threshold (Compte et al., 2003). Therefore, these neurons play the role of pacemakers, or drivers of oscillations. Here we show that in order to make such an oscillator it is unnecessary to change the neuron model or introduce any persistent currents. In our model, we build the oscillation merely by increasing the connection probability between weight-hubs (to about 50%), while keeping the overall connection probability fixed at the value measured in experiments (Lefort et al., 2009).

The return of the cortex from the up-state to the down-state could have several reasons. In one approach, the accumulation or increase of inhibition shuts down the up-state: When the system goes to the up-state, the excitatory population receives excitation from itself and inhibition from an inhibitory population. The system remains in the up-state as long as the excitation dominates the inhibition. However, at some point the inhibition becomes dominant and brings the system back to the down-state (Parga and Abbott, 2007; Melamed et al., 2008). Instead of inhibition, synaptic short-term depression may weaken the excitatory to excitatory synapses during the up-state and cause a reduction of self-excitation received by the excitatory population, by which it can oscillate on its own (Holcman and Tsodyks, 2006). In Compte et al. (2003) and Giugliano et al. (2004) spike-frequency adaptation is responsible for bringing the system back to the down-state. In the up-state, Na+ -activated K+ channels reduce the firing rate of excitatory neurons gradually and the excitatory population loses the amount of self-excitation that is necessary for remaining in the up-state. Consequently it falls back to the down-state. Our model also uses adaptation for switching to the down-state. We have previously built a similar model which uses short-term depression instead of adaptation (Setareh et al., 2014). As shown here in a network of neurons that exhibit spike-frequency adaptation with parameters fitted from experiments, synaptic depression is not necessary, but we do not exclude that synaptic depression plays a role in cortical up-/down-states as well.

### Competition based network models

An important point which distinguishes our work from several previous models (Shpiro et al., 2009; Krishnamurthy et al., 2012) is that there is no competition between the assemblies of weight-hub neurons in our model. In classic competition based models there are two or more populations of excitatory neurons, each trying to become active and suppress the other ones using either direct inhibition or indirectly by exciting an inhibitory population of neurons. The dominant population keeps inhibiting others until it loses its activation by a negative feedback mechanism like short-term depression or spike-frequency adaptation, or until one of the suppressed populations becomes the dominant one by receiving a high amount of noise sufficient to overcome the inhibition. In contrast, in our model assemblies do not compete to win the activation. In contrast to inhibition-dominated networks, in our network, the active assembly helps other assemblies (and non-hubs) to become active by sending excitation more than indirect inhibition. As a consequence of our network parameters, several assemblies can be active simultaneously. Depending on the adaptation state of the assemblies at the time of receiving excitation, the number and order of transitions to the up-state are different. Such different patterns of activations cause different up-state duration in the non-hub and inhibitory neurons.

### Models for producing multistable activity

While our model is not based on competition of assemblies, and several assemblies can be active at the same time, it is also different from clustered network models suggested for producing multistable activity states (Deco et al., 2011; Litwin-Kumar and Doiron, 2012; Mazzucato et al., 2015, 2016). In such models several clusters of neurons are embedded into the population of excitatory neurons. The connection probabilities inside clusters are increased (in Litwin-Kumar and Doiron, 2012 both connection probabilities and synaptic weights are increased) compared to those between clusters. Therefore, each cluster acts as an attractor similar to the assemblies of weight-hub neurons in our model. Clusters receive noisy input and one or several of them become active at a time. The shared inhibitory population sends inhibition to all clusters and limits the number of active clusters. Once one of the quiescent clusters, which also receives noisy input, becomes active, due to shared inhibition, it deactivates one or several clusters which were previously active. This procedure repeats and consequently, each cluster switches between active and inactive states. In this architecture, coincidence of noisy inputs causes a cluster to switch to the active state and shared inhibition switches it back to the inactive state. Therefore, there is no need for a negative feedback mechanism like spike-frequency adaptation. Although the activity of this model looks similar to ours, the functionality is different. In these models, at least one cluster is active at each time during ongoing activity. In fact, a cluster deactivates because another one becomes active—the clusters pass on the activity amongst themselves. Therefore, after a first activation there is at least one active cluster which produces spikes and depolarizes other neurons including non-cluster excitatory neurons (the situation that all clusters become inactive at the same time rarely occurs). In contrast, in the down-state all neurons are silent and have a low membrane potential. Moreover, in the clustered network transitions into and out of the active state are anti-correlated between different assemblies: If one cluster activates, another one typically deactivates, i.e., the number of active clusters is constant most of the times, although we may rarely observe several active assemblies at the same time. In our model, transitions times to up- and down-states are correlated across the network. Moreover, each assembly is able to transition back to the inactive state (the low-point) on its own without need for inhibition or activation of other assemblies. The self-termination ability results from spike-frequency adaptation (see Section Materials and Methods). Therefore, there are time intervals in which all assemblies are inactive and the whole network is silent. In summary, although the clustered architecture successfully reproduces the multistable states during ongoing and evoked activity, it is not suitable to produce up-state/down-state oscillations.

To conclude we would like to highlight the predictive aspects of our study. First, central components of our model are the weight-hub neurons, i.e., those with strong synaptic inputs. Although, there is no direct experimental evidence for the existence of weight-hub neurons, we introduce this concept here as a prediction. Yet, our demonstrations that model networks using weight-hubs display biologically plausible dynamics, and explain cortical phenomena, may be considered as an indication of weight-hub existence. Second, on top of that, we predict that weight-hub neurons in L5 form an assembly of strongly connected cells, while the weight-hubs are sparsely connected in L2/3. Third, the up-down state transitions of neurons within a weight-hub assembly are more regular than that in the majority of other neurons. All of these predictions can be tested in future experiments.

## Author contributions

HS designed and performed the research and wrote the manuscript. MD designed the research, analyzed the results and wrote the manuscript. CP analyzed the results and wrote the manuscript. WG analyzed the results and wrote the manuscript.

### Conflict of interest statement

The authors declare that the research was conducted in the absence of any commercial or financial relationships that could be construed as a potential conflict of interest.

## References

[B1] Amit D. J. Brunel N. (1997). Model of global spontaneous activity and local structured activity during delay periods in the cerebral cortex. Cereb. Cortex 7, 237–252. 10.1093/cercor/7.3.237 9143444

[B2] Avermann M. Tomm C. Mateo C. Gerstner W. Petersen C. C. H. (2012). Microcircuits of excitatory and inhibitory neurons in layer 2/3 of mouse barrel cortex. J. Neurophysiol. 107, 3116–3134. 10.1152/jn.00917.2011 22402650

[B3] Bazhenov M. Timofeev I. Steriade M. Sejnowski T. J. (2002). Model of thalamocortical slow-wave sleep oscillations and transitions to activated states. J. Neurosci. 22, 8691–8704. 12351744 10.1523/JNEUROSCI.22-19-08691.2002 PMC6757797

[B4] Beltramo R. D'Urso G. Maschio M. D. Farisello P. Bovetti S. Clovis Y.. (2013). Layer-specific excitatory circuits differentially control recurrent network dynamics in the neocortex. Nat. Neurosci. 16, 227–234. 10.1038/nn.3306 23313909

[B5] Binzegger T. Douglas R. J. Martin K. A. (2004). A quantitative map of the circuit of cat primary visual cortex. J. Neurosci. 24, 8441–8453. 10.1523/JNEUROSCI.1400-04.2004 15456817 PMC6729898

[B6] Bonifazi P. Goldin M. Picardo M. A. Jorquera I. Cattani A. Bianconi G.. (2009). GABAergic hub neurons orchestrate synchrony in developing hippocampal networks. Science 326, 1419–1424. 10.1126/science.1175509 19965761

[B7] Bon-Jego M. L. Yuste R. (2007). Persistently active, pacemaker-like neurons in neocortex. Front. Neurosci. 1, 123–129. 10.3389/neuro.01.1.1.009.2007 18982123 PMC2518052

[B8] Brunel N. (2000). Dynamics of sparsely connected networks of excitatory and inhibitory spiking neurons. J. Comput. Neurosci. 8, 183–208. 10.1023/A:1008925309027 10809012

[B9] Bullmore E. Sporns O. (2009). Complex brain networks: graph theoretical analysis of structural and functional systems. Nat. Rev. Neurosci. 10, 186–198. 10.1038/nrn2575 19190637

[B10] Chapeton J. Fares T. LaSota D. Stepanyants A. (2012). Efficient associative memory storage in cortical circuits of inhibitory and excitatory neurons. Proc. Natl. Acad. Sci. U.S.A. 109, E3614–E3622. 10.1073/pnas.1211467109 23213221 PMC3529061

[B11] Chauvette S. Volgushev M. Timofeev I. (2010). Origin of active states in local neocortical networks during slow sleep oscillation. Cereb. Cortex 20, 2660–2674. 10.1093/cercor/bhq009 20200108 PMC2951844

[B12] Compte A. Sanchez-Vives M. V. McCormick D. A. Wang X. J. (2003). Cellular and network mechanisms of slow oscillatory activity (<1 hz) and wave propagations in a cortical network model. J. Neurophysiol. 89, 2707–2725. 10.1152/jn.00845.2002 12612051

[B13] Cossart R. Aronov D. Yuste R. (2003). Attractor dynamics of network up states in the neocortex. Nature 423, 283–288. 10.1038/nature01614 12748641

[B14] Cowan R. L. Wilson C. J. (1994). Spontaneous firing patterns and axonal projections of single corticostriatal neurons in the rat medial agranular cortex. J. Neurophysiol. 71, 17–32. 8158226 10.1152/jn.1994.71.1.17

[B15] Deco G. Jirsa V. K. McIntosh A. R. (2011). Emerging concepts for the dynamical organization of resting- state activity in the brain. Nat. Rev. Neurosci. 12, 43–56. 10.1038/nrn2961 21170073

[B16] Ecker A. S. Berens P. Keliris G. A. Bethge M. Logothetis N. K. Tolias A. S. (2010). Decorrelated neuronal firing in cortical microcircuits. Science 27, 584–587. 10.1126/science.1179867 20110506

[B17] Feldmeyer D. Egger V. Lübke J. Sakmann B. (1999). Reliable synaptic connections between pairs of excitatory layer 4 neurones within a single barrel of developing rat somatosensory cortex. J. Physiol. 521, 169–190. 10.1111/j.1469-7793.1999.00169.x 10562343 PMC2269646

[B18] Feldmeyer D. Lübke J. Sakmann B. (2006). Efficacy and connectivity of intracolumnar pairs of layer 2/3 pyramidal cells in the barrel cortex of juvenile rats. J. Physiol. 575, 583–602. 10.1113/jphysiol.2006.105106 16793907 PMC1819447

[B19] Feldmeyer D. Lübke J. Silver R. A. Sakmann B. (2002). Synaptic connections between layer 4 spiny neurone-layer 2/3 pyramidal cell pairs in juvenile rat barrel cortex: physiology and anatomy of interlaminar signalling within a cortical column. J. Physiol. 538, 803–822. 10.1113/jphysiol.2001.012959 11826166 PMC2290091

[B20] Feldt S. Bonifazi P. Cossart R. (2011). Dissecting functional connectivity of neuronal microcircuits: experimental and theoretical insights. Trends Neurosci. 34, 225–236. 10.1016/j.tins.2011.02.007 21459463

[B21] Fino E. Yuste R. (2011). Dense inhibitory connectivity in neocortex. Neuron 69, 1188–1203. 10.1016/j.neuron.2011.02.025 21435562 PMC3086675

[B22] Fourcaud-Trocmé N. Hansel D. Vreeswijk C. V. Brunel N. (2003). How spike generation mechanisms determine the neuronal response to fluctuating inputs. J. Neurosci. 23, 11628–11640. 14684865 10.1523/JNEUROSCI.23-37-11628.2003 PMC6740955

[B23] Frick A. Feldmeyer D. Helmstaedter M. Sakmann B. (2008). Monosynaptic connections between pairs of L5a pyramidal neurons in columns of juvenile rat somatosensory cortex. Cereb. Cortex 18, 397–406. 10.1093/cercor/bhm074 17548800

[B24] Fucke T. Suchanek D. Nawrot M. P. Seamari Y. Heck D. H. Aertsen A.. (2011). Stereotypical spatiotemporal activity patterns during slow-wave activity in the neocortex. J. Neurophysiol. 106, 3035–3044. 10.1152/jn.00811.2010 21849616

[B25] Gerstner W. (2000). Population dynamics of spiking neurons: fast transients, asynchronous states, and locking. Neural Comput. 12:43–89. 10.1162/089976600300015899 10636933

[B26] Gerstner W. Kistler W. M. Naud R. Paninski L. (2014). Neuronal Dynamics: From Single Neurons to Networks and Models of Cognition. Lausanne: Cambridge University Press.

[B27] Ghorbani M. Mehta M. Bruinsma R. Levine A. J. (2012). Nonlinear-dynamics theory of up-down transitions in neocortical neural networks. Phys. Rev. E 85:021908. 10.1103/physreve.85.021908 22463245

[B28] Giugliano M. Camera G. L. Fusi S. Senn W. (2008). The response of cortical neurons to *in vivo*-like input current: theory and experiment: II). time-varying and spatially distributed inputs. Biol. Cybern. 99, 303–318. 10.1007/s00422-008-0270-9 19011920

[B29] Giugliano M. Darbon P. Arsiero M. Lüscher H. R. Streit J. (2004). Single-neuron discharge properties and network activity in dissociated cultures of neocortex. J. Neurophysiol. 92, 977–996. 10.1152/jn.00067.2004 15044515

[B30] Goodman D. Brette R. (2008). Brian: a simulator for spiking neural networks in python. Front. Neuroinform. 2:5. 10.1186/1471-2202-9-s1-p92 19115011 PMC2605403

[B31] Hebb D. O. (1949). The Organisation of Behaviour: A Neuropsychological Theory. New York, NY: Wiley.

[B32] Hertäg L. Durstewitz D. Brunel N. (2014). Analytical approximations of the firing rate of an adaptive exponential integrate-and-fire neuron in the presence of synaptic noise. Front. Comput. Neurosci. 8:116. 10.3389/fncom.2014.00116 25278872 PMC4167001

[B33] Hill S. Tononi G. (2005). Modeling sleep and wakefulness in the thalamocortical system. J. Neurophysiol. 93, 1671–1698. 10.1152/jn.00915.2004 15537811

[B34] Holcman D. Tsodyks M. (2006). The emergence of up and down states in cortical networks. PLoS Comput. Biol. 2:e23. 10.1371/journal.pcbi.0020023 16557293 PMC1409813

[B35] Hromádka T. DeWeese M. R. Zador A. M. (2008). Sparse representation of sounds in the unanesthetized auditory cortex. PLoS Biol. 6:e16. 10.1371/journal.pbio.0060016 18232737 PMC2214813

[B36] Hu Y. Trousdale J. Josicì K. Shea-Brown E. (2013). Motif statistics and spike correlations in neuronal networks. J. Stat. Mech. Theor. Exp. 03:P03012 10.1088/1742-5468/2013/03/p03012

[B37] Hu Y. Trousdale J. Josicì K. Shea-Brown E. (2014). Local paths to global coherence: cutting networks down to size. Phys. Rev. E 89:032802. 10.1103/physreve.89.032802 24730894

[B38] Iyer R. Menon V. Buice M. Koch C. Mihalas S. (2013). The influence of synaptic weight distribution on neuronal population dynamics. PLoS Comput. Biol. 9:e1003248. 10.1371/journal.pcbi.1003248 24204219 PMC3808453

[B39] Jahnke S. Memmesheimer R. M. Timme M. (2014). Hub-activated signal transmission in complex networks. Phys. Rev. E 89:030701. 10.1103/physreve.89.030701 24730779

[B40] Jolivet R. Rauch A. Lüscher H. R. Gerstner W. (2006). Predicting spike timing of neocortical pyramidal neurons by simple threshold models. J. Comput. Neurosci. 21, 35–49. 10.1007/s10827-006-7074-5 16633938

[B41] Kampa B. M. Letzkus J. J. Stuart G. J. (2006). Cortical feed-forward networks for binding different streams of sensory information. Nat. Neurosci. 9, 1472–1473. 10.1038/nn1798 17099707

[B42] Klinshov V. V. Teramae J. N. Nekorkin V. I. Fukai T. (2014). Dense neuron clustering explains connectivity statistics in cortical microcircuits. PLoS ONE 9:e94292. 10.1371/journal.pone.0094292 24732632 PMC3986068

[B43] Ko H. Hofer S. B. Pichler B. Buchanan K. A. Sjöström P. J. Mrsic-Flogel T. D. (2011). Functional specificity of local synaptic connections in neocortical networks. Nature 473, 87–91. 10.1038/nature09880 21478872 PMC3089591

[B44] Koulakov A. A. Hromadka T. Zador A. M. (2009). Correlated connectivity and the distribution of firing rates in the neocortex. J. Neurosci. 29, 3685–3694. 10.1523/JNEUROSCI.4500-08.2009 19321765 PMC2784918

[B45] Krishnamurthy P. Silberberg G. Lansner A. (2012). A cortical attractor network with Martinotti cells driven by facilitating synapses. PLoS ONE 7:e30752. 10.1371/journal.pone.0030752 22523533 PMC3327695

[B46] La Camera G. Rauch A. Lüscher H. R. Senn W. Fusi S. (2004). Minimal models of adapted neuronal response to *in vivo*-like input currents. Neural Comput. 16, 2101–2124. 10.1162/0899766041732468 15333209

[B47] Laing C. R. Chow C. C. (2002). A spiking neuron model for binocular rivalry. J. Comput. Neurosci. 12, 39–53. 10.1023/A:1014942129705 11932559

[B48] Lampl I. Reichova I. Ferster D. (1999). Synchronous membrane potential fluctuations in neurons of the cat visual cortex. Neuron 22, 361–374. 10069341 10.1016/s0896-6273(00)81096-x

[B49] Lefort S. Tomm C. Sarria J. C. F. Petersen C. C. (2009). The excitatory neuronal network of the C2 barrel column in mouse primary somatosensory cortex. Neuron 61, 301–316. 10.1016/j.neuron.2008.12.020 19186171

[B50] Litwin-Kumar A. Doiron B. (2012). Slow dynamics and high variability in balanced cortical networks with clustered connections. Nat. Neurosci. 15, 1498–1505. 10.1038/nn.3220 23001062 PMC4106684

[B51] Luccioli S. Ben-Jacob E. Barzilai A. Bonifazi P. Torcini A. (2014). Clique of functional hubs orchestrates population bursts in developmentally regulated neural networks. PLoS Comput. Biol. 10:e1003823. 10.1371/journal.pcbi.1003823 25255443 PMC4177675

[B52] Markram H. Muller E. Ramaswamy S. Reimann M. W. Abdellah M. Sanchez C. A.. (2015). Reconstruction and simulation of neocortical microcircuitry. Cell 163, 456–492. 10.1016/j.cell.2015.09.029 26451489

[B53] Markram H. Toledo-Rodriguez M. Wang Y. Gupta A. Silberberg G. Caizhi W. (2004). Interneurons of the neocortical inhibitory system. Nat. Rev. Neurosci. 5, 793–807. 10.1038/nrn1519 15378039

[B54] Mazzucato L. Fontanini A. La Camera G. (2015). Dynamics of multistable states during ongoing and evoked cortical activity. J. Neurosci. 35, 8214–8231. 10.1523/JNEUROSCI.4819-14.2015 26019337 PMC4444543

[B55] Mazzucato L. Fontanini A. La Camera G. (2016). Stimuli reduce the dimensionality of cortical activity. Front. Syst. Neurosci. 10:11. 10.3389/fnsys.2016.00011 26924968 PMC4756130

[B56] McDonnell M. Ward L. (2014). Small modifications to network topology can induce stochastic bistable spiking dynamics in a balanced cortical model. PLoS ONE 9:e88254. 10.1371/journal.pone.0088254 24743633 PMC3990528

[B57] Melamed O. Barak O. Silberberg G. Markram H. Tsodyks M. (2008). Slow oscillations in neural networks with facilitating synapses. J. Comput. Neurosci. 25, 308–316. 10.1007/s10827-008-0080-z 18483841

[B58] Mensi S. Naud R. Pozzorini C. Avermann M. Petersen C. C. Gerstner W. (2012). Parameter extraction and classification of three cortical neuron types reveals two distinct adaptation mechanisms. J. Neurophysiol. 107, 1756–1775. 10.1152/jn.00408.2011 22157113

[B59] Moreno-Bote R. Rinzel J. Rubin N. (2007). Noise-induced alternations in an attractor network model of perceptual bistability. J. Neurophysiol. 98, 1125–1139. 10.1152/jn.00116.2007 17615138 PMC2702529

[B60] Okun M. Steinmetz N. Cossell L. Iacaruso M. Ko H. Barthó P.. (2015). Diverse coupling of neurons to populations in sensory cortex. Nature 521, 511–515. 10.1038/nature14273 25849776 PMC4449271

[B61] Parga N. Abbott L. F. (2007). Network model of spontaneous activity exhibiting synchronous transitions between up and down states. Front. Neurosci. 1, 57–66. 10.3389/neuro.01.1.1.004.2007 18982119 PMC2570086

[B62] Perin R. Berger T. K. Markram H. (2011). A synaptic organizing principle for cortical neuronal groups. Proc. Natl. Acad. Sci. U.S.A. 108, 5419–5424. 10.1073/pnas.1016051108 21383177 PMC3069183

[B63] Pernice V. Deger M. Cardanobile S. Rotterf S. (2013). The relevance of network micro-structure for neural dynamics. Front. Comput. Neurosci. 7:72. 10.3389/fncom.2013.00072 23761758 PMC3671286

[B64] Petersen C. C. H. Hahn T. T. Mehta M. Grinvald A. Sakmann B. (2003). Interaction of sensory responses with spontaneous depolarization in layer 2/3 barrel cortex. Proc. Natl. Acad. Sci. U.S.A. 100, 13638–13643. 10.1073/pnas.2235811100 14595013 PMC263866

[B65] Pfeffer C. K. Xue M. He M. Josh Z. Scanziani M. (2013). Inhibition of inhibition in visual cortex: the logic of connections between molecularly distinct interneurons. Nat. Neurosci. 16, 1068–1076. 10.1038/nn.3446 23817549 PMC3729586

[B66] Pillow J. W. Shlens J. Paninski L. Sher A. Litke A. M. Chichilnisky E.. (2008). Spatio- temporal correlations and visual signalling in a complete neuronal population. Nature 454, 995–999. 10.1038/nature07140 18650810 PMC2684455

[B67] Potjans T. C. Diesmann M. (2014). The cell-type specific cortical microcircuit: relating structure and activity in a full-scale spiking network model. Cereb. Cortex 24, 785–806. 10.1093/cercor/bhs358 23203991 PMC3920768

[B68] Poulet J. F. Fernandez L. M. Crochet S. Petersen C. C. (2012). Thalamic control of cortical states. Nat. Neurosci. 15, 370–372. 10.1038/nn.3035 22267163

[B69] Pozzorini C. Mensi S. Hagens O. Naud R. Koch C. Gerstner W. (2015). Automated high-throughput characterization of single neurons by means of simplified spiking models. PLoS Comput. Biol. 11:e1004275. 10.1371/journal.pcbi.1004275 26083597 PMC4470831

[B70] Pozzorini C. Naud R. Mensi S. Gerstner W. (2013). Temporal whitening by power-law adaptation in neocortical neurons. Nat. Neurosci. 16, 942–948. 10.1038/nn.3431 23749146

[B71] Prettejohn B. J. Berryman M. J. McDonnell M. D. (2011). Methods for generating complex networks with selected structural properties for simulations: a review and tutorial for neuroscientists. Front. Comput. Neurosci. 5:11. 10.3389/fncom.2011.00011 21441986 PMC3059456

[B72] Reich D. S. Mechler F. Victor J. D. (2001). Independent and redundant information in nearby cortical neurons. Science 294, 2566–2568. 10.1126/science.1065839 11752580

[B73] Richardson M. J. (2007). Firing-rate response of linear and nonlinear integrate-and-fire neurons to modulated current-based and conductance-based synaptic drive. Phys. Rev. E 76:021919. 10.1103/physreve.76.021919 17930077

[B74] Richardson M. J. (2009). Dynamics of populations and networks of neurons with voltage-activated and calcium-activated currents. Phys. Rev. E 80:021928. 10.1103/physreve.80.021928 19792172

[B75] Roxin A. (2011). The role of degree distribution in shaping the dynamics in networks of sparsely connected spiking neurons. Front. Comput. Neurosci. 5:8. 10.3389/fncom.2011.00008 21556129 PMC3058136

[B76] Roxin A. Brunel N. Hansel D. Mongillo G. van Vreeswijk C. (2011). On the distribution of firing rates in networks of cortical neurons. J. Neurosci. 31, 16217–16226. 10.1523/JNEUROSCI.1677-11.2011 22072673 PMC6633220

[B77] Rudolph-Lilith M. Muller L. E. (2014). Aspects of randomness in neural graph structures. Biol. Cybern. 108, 381–396. 10.1007/s00422-014-0606-6 24824724

[B78] Sakata S. Harris K. D. (2009). Laminar structure of spontaneous and sensory-evoked population activity in auditory cortex. Neuron 64, 404–418. 10.1016/j.neuron.2009.09.020 19914188 PMC2778614

[B79] Sanchez-Vives M. V. McCormick D. A. (2000). Cellular and network mechanisms of rhythmic recurrent activity in neocortex. Nat. Neurosci. 3, 1027–1034. 10.1038/79848 11017176

[B80] Sanchez-Vives M. V. Nowak L. G. McCormick D. A. (2000). Membrane mechanisms underlying contrast adaptation in cat area 17 *in vivo*. J. Neurosci. 20, 4267–4285. 10818163 10.1523/JNEUROSCI.20-11-04267.2000 PMC6772627

[B81] Setareh H. Deger M. Gerstner W. (2014). The role of interconnected hub neurons in cortical dynamics. BMC Neurosci. 15(Suppl. 1):P158 10.1186/1471-2202-15-S1-P158

[B82] Shpiro A. Moreno-Bote R. Rubin N. Rinzel J. (2009). Balance between noise and adaptation in competition models of perceptual bistability. J. Comput. Neurosci. 27, 37–54. 10.1007/s10827-008-0125-3 19125318 PMC2913428

[B83] Song S. Sjostrom P. J. Reigl M. Nelson S. Chklovskii D. B. (2005). Highly nonrandom features of synaptic connectivity in local cortical circuits. PLoS Biol. 3:e68. 10.1371/journal.pbio.0030068 15737062 PMC1054880

[B84] Steriade M. Nunez A. Amzica F. (1993). A novel slow (< 1 Hz) oscillation of neocortical neurons *in vivo*: depolarizing and hyperpolarizing components. J. Neurosci. 13, 3252–3265. 8340806 10.1523/JNEUROSCI.13-08-03252.1993 PMC6576541

[B85] Stern E. A. Kincaid A. E. Wilson C. J. (1997). Spontaneous subthreshold membrane potential fluctuations and action potential variability of rat corticostriatal and striatal neurons *in vivo*. J. Neurophysiol. 77, 1697–1715. 9114230 10.1152/jn.1997.77.4.1697

[B86] Timofeev I. Grenier F. Bazhenov M. Sejnowski T. J. Steriade M. (2000). Origin of slow cortical oscillations in deafferented cortical slabs. Cereb. Cortex 10, 1185–1199. 10.1093/cercor/10.12.1185 11073868

[B87] Tomm C. Avermann M. Petersen C. C. Gerstner W. Vogels T. P. (2014). Connection-type specific biases make random network models consistent with cortical recordings. J. Neurophysiol. 112, 1801–1814. 10.1152/jn.00629.2013 24944218 PMC4200009

[B88] Vasquez J. Houweling A. Tiesinga P. (2013). Simultaneous stability and sensitivity in model cortical networks is achieved through anti-correlations between the in-and out-degree of connectivity. Front. Comput. Neurosci. 7:156. 10.3389/fncom.2013.00156 24223550 PMC3819735

[B89] Vijayan S. Hale G. J. Moore C. I. Brown E. N. Wilson M. (2010). Activity in the barrel cortex during active behavior and sleep. J. Neurophysiol. 103, 2074–2084. 10.1152/jn.00474.2009 20164403 PMC2853270

[B90] Yassin L. Benedetti B. L. Jouhanneau J. S. Wen J. A. Poulet J. F. Barth A. L. (2010). An embedded sub- network of highly active neurons in the neocortex. Neuron 68, 1043–1050. 10.1016/j.neuron.2010.11.029 21172607 PMC3022325

[B91] Yoshimura Y. Dantzker J. L. Callaway E. M. (2005). Excitatory cortical neurons form fine-scale functional networks. Nature 433, 868–873. 10.1038/nature03252 15729343

